# Redox System Dysfunction as a Key Mechanism in Autism Spectrum Disorder Pathogenesis

**DOI:** 10.3390/ijms26209850

**Published:** 2025-10-10

**Authors:** Clarissa Aires de Oliveira, Eugenio Luigi Iorio, Foued Salmen Espíndola

**Affiliations:** 1 Biochemistry and Molecular Biology Laboratory, Institute of Biotechnology, Federal University of Uberlandia, Uberlandia 38405-319, MG, Brazil; 2 Program of Post-Graduation in Health Science, Federal University of Uberlandia, Uberlandia 38405-319, MG, Brazil

**Keywords:** Autism Spectrum Disorder, neuroinflammation, neurodevelopmental vulnerability, redox system, oxidative stress, mitochondrial dysfunction

## Abstract

Autism Spectrum Disorder (ASD) is a complex and multifactorial neurodevelopmental condition whose pathogenesis remains only partially elucidated. Earlier accounts of oxidative stress in ASD often relied on the reductive paradigm of an imbalance between oxidants and antioxidants. In contrast, this narrative review, based on a systematic examination of 1102 publications indexed in scientific databases from 2002 to July 2025, reframes the discussion in terms of redox system dysfunction, a broader and more integrative construct. Here, reactive oxidant species, molecular targets, and reducing/antioxidant counterparts are considered elements of a dynamic circuitry whose maladaptation progressively undermines homeostasis. The sequence of events unfolds in three stages. The first is primary redox dysfunction, manifesting as alterations in metabolic, signaling, and defense pathways. From this disturbance, a second stage arises, marked by functional derailment of cellular compartments—from membranes and cytosol to organelles and nuclei—including mitochondrial and peroxisomal deficits. Ultimately, a third stage emerges, defined by neurodevelopmental alterations such as impaired neurotransmission, synaptic dysfunction, abnormal plasticity, morphogenetic defects, neuroinflammation, and gut–brain–microbiota disarrangements. This progression situates the redox system as a central hub at the interface between human cells and the microbiota, resonating with the ecological and evolutionary principles of the holobiont and the One Health framework. By weaving dispersed evidence into a coherent perspective, this review advances beyond previous analyses, offering a unifying paradigm that connects biochemical dysfunction to clinical heterogeneity in ASD and opens new directions for interdisciplinary research.

## 1. Introduction

Autism Spectrum Disorder (ASD) is a multifactorial neurodevelopmental condition defined by social-communication deficits and restricted behaviors, with prevalence now estimated at 1 in 36 children in the United States [1,2,3,4]. Its etiology involves a complex interplay of genetic, epigenetic, and environmental influences, yet converging evidence highlights a central biochemical vulnerability: redox imbalance [5,6,7].

More than 1000 genes have been implicated in ASD, many of which affect synaptic function, chromatin remodeling, and developmental signaling, with several intersecting pathways sensitive to oxidative regulation. On the other hand, epigenetic modifications and environmental factors such as maternal immune activation (MIA) or pollutant exposure further challenge redox homeostasis [5,8]. Therefore, traditionally attributed to disrupted synaptogenesis, connectivity, and neuroinflammation [9,10], ASD is now increasingly framed within a redox-centered model where oxidative stress (OS) integrates diverse pathogenic inputs [11,12,13,14].

In our view, OS is not a static imbalance but the dysfunctional output of the redox system—a conserved triad of reactive oxidant species (ROS), molecular targets, and reducing/antioxidant (AOX) agents that normally orchestrates metabolic, signaling, and defensive pathways, through exchange of single electrons [15,16,17,18]. In ASD, we hypothesize that this adaptive circuitry collapses into chronic oxidative distress, reshaping neurodevelopmental trajectories [19,20].

To develop this perspective, we conducted a targeted literature search across PubMed, Scopus, ResearchGate, Google Scholar, and Web of Science, identifying 1102 articles published between 2002 and July 2025. Sources were selected to cover genetic, epigenetic, environmental, neurobiological, and biochemical aspects of ASD, with studies limited to biomarkers or nutraceutical interventions intentionally excluded. The goal was not to provide a protocol-driven systematic review but rather a narrative synthesis, integrating heterogeneous findings into an interpretative framework that highlights the centrality of redox system dysfunction in ASD pathogenesis.

Before expanding this hypothesis, we briefly revisit how “oxidative stress” is currently conceptualized in the literature.

## 2. Reconsidering the Concept of Oxidative Stress: Some Possible Shortcomings

Despite extensive research, the concept of OS in ASD remains ambiguous, limiting data interpretation and clinical translation [19,21,22]. Most studies still rely on Halliwell’s classical paradigm—“an imbalance between reactive oxygen species production and antioxidant defenses, disrupting redox signaling and/or causing molecular damage.” While historically useful, this model oversimplifies the compartmentalized and heterogeneous redox biology of the brain [23,24]. Crucially, the distinction between oxidative eustress—a physiological adaptive response—and oxidative distress—a maladaptive state—is often overlooked, with ASD research trapped in the oxidant–antioxidant dichotomy [23,25,26].

Critical analysis reveals recurrent misconceptions. A common error is treating the electron as the unit of exchange in redox reactions, rather than the reducing equivalent unit [27]. Similarly, OS is often conflated with all redox reactions, when only single-electron transfer defines the process [28,29]. Reactive oxidant species are usually limited to oxygen- and nitrogen-centered radicals, neglecting carbon-, sulfur-, and chlorine-centered species, which also contribute to ASD pathogenesis [19,20]. Superoxide is frequently mischaracterized as a reactive oxidant species, obscuring the nuanced role of superoxide dismutases (SOD) [25,30]. Free radicals are routinely portrayed as universally “highly reactive oxidants,” disregarding their potential to act as oxidants or reductants depending on redox potential, and their variable stability [19,20]. Definitions often exclude biological targets, reducing redox dynamics to a simplistic battle between oxidants and AOX [15,31]. This “good versus bad” dichotomy also blurs the distinction between reducing species and AOX, overlapping but not identical categories [21,22] Compartmentalization across tissues, cell types, and organelles is largely neglected, fostering misleading generalizations [32]. Additional misconceptions concern the nervous system. It is often depicted as strictly aerobic and oxygen-dependent, although neural stem cells thrive in hypoxic niches, challenging traditional assumptions [32,33,34]. Moreover, the nervous system is usually presented as a producer of reactive oxidants, with insufficient recognition of its vulnerability as a primary target [35,36].

Altogether, these inaccuracies highlight the urgent need to refine the OS framework in ASD. A revised model must distinguish eustress from distress, acknowledge chemical specificity and spatial compartmentalization, and integrate biological targets [37,38]. Such a framework is essential for clarifying pathogenetic mechanisms, improving diagnostic strategies, and guiding innovative therapeutic approaches [25,37,39,40].

## 3. Rethinking the Starting Point in ASD Pathophysiology: From Oxidative Stress to Redox System

**Figure 1:**
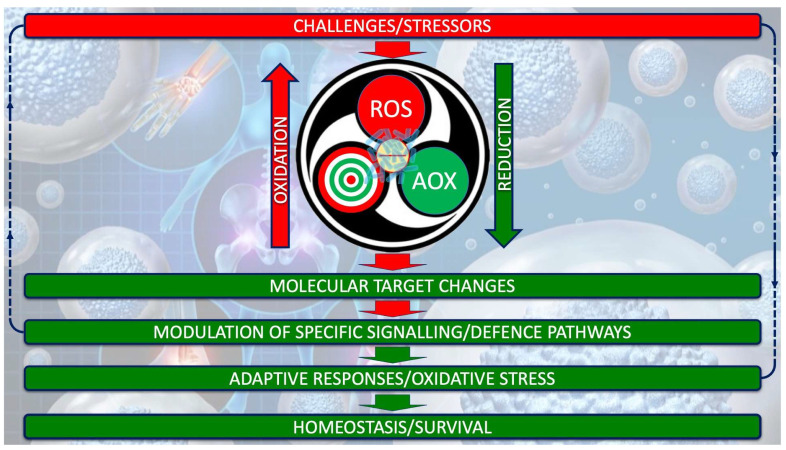
Redox system architecture. The redox system is not a mere oxidant/antioxidant counterbalance but a highly dynamic biochemical network that finely regulates single-electron (the negative electric charge at the center of the system) transfers between reactive oxidant species (ROS), molecular targets, and reducing/AOX agents. This orchestrated exchange interfaced with gut microbiota (superimposed in transparency on the electric charge) underpins the adaptive capacity of cells to sense, process, and resolve oxidative challenges, thereby safeguarding redox homeodynamics.

Understanding OS in ASD requires moving beyond the simplistic imbalance between oxidants and antioxidants, toward a systems-based view of redox biology [15,31,34]. Oxidative stress is now seen as an adaptive response within the redox system, which operates through single reducing-equivalent exchanges involving three interdependent actors: an oxidant species, a molecular target, and a reducing/AOX species [16,17,18]. This triad regulates metabolism, signaling, and defense under both physiological and pathological conditions, as depicted in Figure 1.

Reactive oxidant species extend beyond oxygen- and nitrogen-centered radicals (reactive nitrogen species, RNS) to include sulfur-, carbon-, and chlorine-centered species, each with distinct reactivities (Table 1) [15,41,42].

Among oxygen-centered species, hydrogen peroxide (H_2_O_2_), with its longer half-life, often serves as a signaling mediator, whereas hydroxyl radicals or hypochlorite exert immediate destructive effects (Table 2) [41,42].

Molecular targets include lipids, proteins, and nucleic acids: polyunsaturated fatty acids (PUFA) are highly susceptible to peroxidation, while protein thiols act as redox switches controlling enzymes and transcription factors [15,43,44,45,46,47,48].

**Figure 2:**
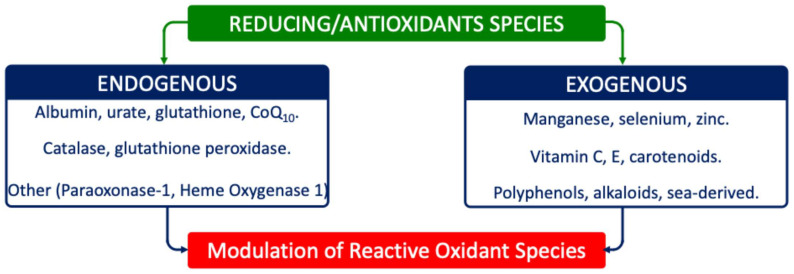
Main reducing/AOX species involved in redox system. Reducing/AOX species include either endogenous or exogenous compounds/enzymes.

Reducing agents such as glutathione (GSH), tocopherols, and ascorbate buffer these modifications; reversible changes can be corrected, but irreversible ones, such as lipid hydroperoxides, demand enzymatic detoxification (Figure 2) [49,50].

**Figure 3:**
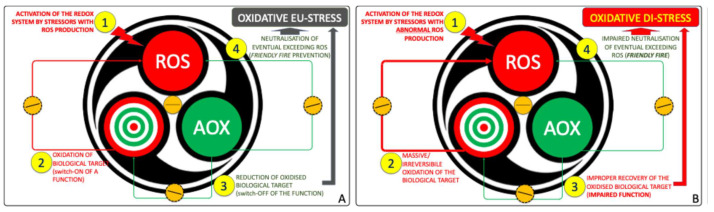
The redox system as manager of OS. (**A**) Under physiological conditions, (1) controlled bursts of Reactive Oxidant Species (ROS) act as signals, (2) transiently oxidizing molecular targets and modulating their function. (3) Antioxidants (AOX) then restore redox balance, while (4) surplus ROS are neutralized, establishing a self-limiting cycle of adaptive oxidative eustress compatible with cellular fitness. (**B**) When this circuitry is dysregulated, (1) ROS production escapes control, (2) causing irreversible oxidation of key and off-target molecules. (3) The antioxidant arm fails to re-establish equilibrium, and (4) the system shifts from eustress to oxidative distress, a hallmark of redox system failure.

This architecture sustains both physiological eustress and pathological distress (Figure 3) [34]. In health, eustress underlies essential processes—respiration, motility, immune defense, excitability, and synaptic plasticity—critical for neurodevelopment (Figure 3A) [26,41,46]. When regulation fails, due to genetic predisposition, environmental exposures, or metabolic deficits, the system shifts toward oxidative distress, defined by excess reactive species, insufficient AOX responses, and cumulative molecular damage (Figure 3B) [16,17,18,23,34,43,51].

The nervous system exemplifies this vulnerability. Despite its small mass, it consumes a disproportionate share of oxygen, predisposing to oxidant overproduction from mitochondria, nicotinamide adenine dinucleotide phosphate (reduced form) (NADPH) oxidases, nitric oxide synthases (NOS), and monoamine oxidases (MAO) [35,52,53,54,55,56,57]. Neural membranes, rich in PUFA, are particularly sensitive, while antioxidant reserves are modest [58,59,60,61]. Moreover, neurons, astrocytes, and microglia possess distinct redox profiles shaped by vascular supply and barrier systems, making the brain both a major generator and a prime target of oxidative imbalance [62,63,64,65].

Genetic and epigenetic backgrounds further modulate susceptibility, as do environmental agents such as pollutants, drugs, or stress [66,67,68]. In predisposed contexts, these triggers precipitate collapse into oxidative distress. Within the central nervous system (CNS), this maladaptive state fosters microglial hyperactivation, neuroinflammation, and impaired synaptic morphogenesis, redirecting developmental trajectories toward atypical circuits typical of ASD [69,70].

Thus, OS is indispensable for physiology but becomes pathogenic when unresolved. Recognizing oxidative distress as a core ASD risk factor underscores the need for biomarkers that capture its spatial and temporal dynamics and supports the hypothesis that redox dysfunction drives neurodevelopmental divergence [37,51,71].

**Figure 4:**
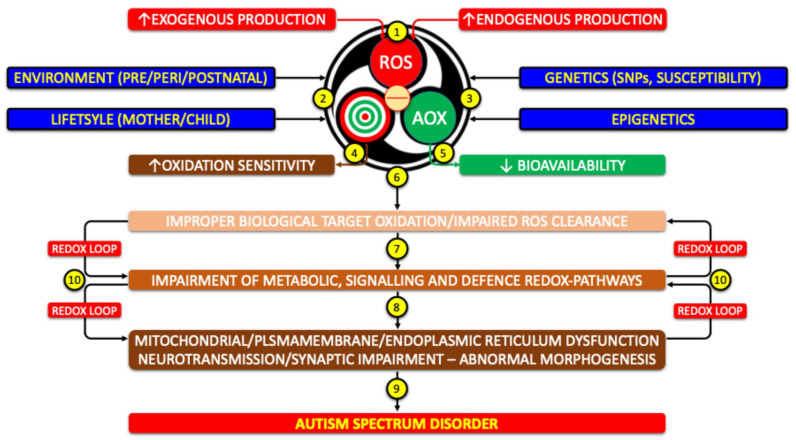
Potential role of redox system dysfunction in the pathogenesis of autism (1) Excess production of ROS, of endogenous or exogenous origin, activates the redox system. (2) Environmental and lifestyle factors and (3) genetic/epigenetic background modulate this response. (4) Increased susceptibility of molecular targets and/or (5) reduced AOX availability trigger (6) a chain reaction. (7) Altered metabolic, signaling, and defense pathways lead to (8) secondary dysfunctions, such as mitochondrial impairment and disruption of the microbiota–gut–brain–mitochondria axis. These changes (9) drive neurodevelopmental alterations consistent with ASD. (10) The process is sustained by self-perpetuating feedback loops of redox dysfunction. (SNPs: Single Nucleotide Polymorphisms).

In our hypothesis, depicted in Figure 4, redox system dysfunction in autism arises from excess ROS, genetic–epigenetic vulnerability of molecular targets, and limited antioxidant performances. This imbalance alters signaling, metabolism, and defense pathways, leading to multiple cell function failure. Feedback loops perpetuate oxidative–nitrosative stress, driving neurodevelopmental abnormalities consistent with ASD.

## 4. Reactive Oxidant Species Levels Are Increased in ASD

In ASD, ROS arise from both excessive production and insufficient neutralization, driven by environmental insults, metabolic imbalance, dysbiosis, and chronic inflammation [7,11,12,51,72,73]. Their pathogenicity depends not only on the species involved but also on mechanisms of generation and the developmental stage at which they occur, being especially harmful in cortical and cerebellar microenvironments during sensitive windows [62,74,75,76]. Reactive oxidant species thus act as both initiators and amplifiers of neurobiological dysfunction, reinforcing maladaptive cycles. This evidence positions redox imbalance as a central driver of ASD and underscores the importance of early recognition and personalized strategies to mitigate long-term neurodevelopmental impact [19,37,71].

### 4.1. Increased Production of ROS by Direct Mechanisms in ASD

**Figure 5:**
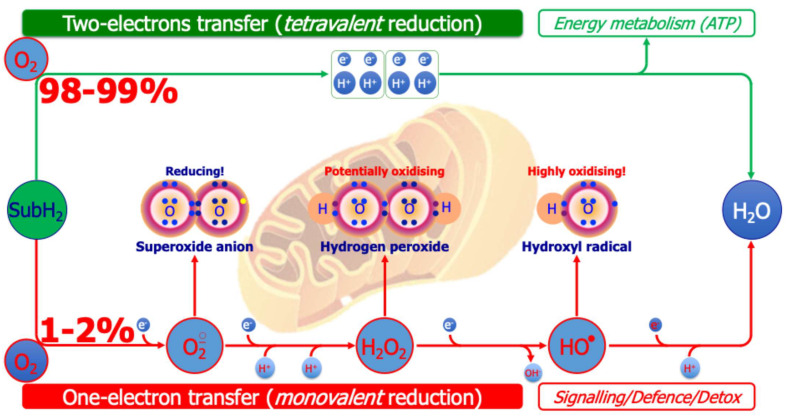
Generation of oxygen-centered reactive species from the mitochondrial respiratory chain. A total of 98–99% of inspired oxygen receives reducing equivalents removed from various metabolic substrates (e.g., fatty acids, glucose) in pairs (tetravalent reduction), generating ATP (top of figure). The remaining 1–2% of inspired oxygen, however, receives reducing equivalents removed from various substrates through one-electron transfer reactions (monovalent reduction), generating oxygen-centered reactive species (bottom of figure), exploited for signaling or defense/detoxification purposes.

Mitochondrial oxidative phosphorylation and dopamine autoxidation are major endogenous ROS sources in ASD [77]. In the respiratory chain, a small fraction of oxygen escapes the four-electron reduction for ATP synthesis, generating superoxide, hydrogen peroxide, and hydroxyl radicals [78,79]. Given that the brain consumes nearly 20% of body oxygen despite its small mass, this leakage has disproportionate consequences, making neural tissue particularly vulnerable (Figure 5) [35,78,79].

Dopamine autoxidation in substantia nigra, striatum, and limbic regions generates quinones and ROS that modify cysteine residues, impair mitochondria, activate microglia, and damage vessels. These events translate into impaired dopaminergic signaling, disruption of synaptic plasticity, and chronic neuroinflammation, contributing to ASD features such as social deficits and stereotypies [78,79,80,81,82,83,84,85].

### 4.2. Increased Production of ROS by Indirect/Catalytic Mechanisms in ASD

#### 4.2.1. Increased Production of ROS by Inorganic Catalysts

Iron and copper catalyze the conversion of hydrogen peroxide into hydroxyl radicals via Fenton and Haber–Weiss reactions [15,24,86,87]. In ASD, impaired ferritin buffering, neuroinflammation, and mitochondrial dysfunction increase free iron [29], amplifying lipid peroxidation and favoring ferroptosis, which destabilizes membranes and synaptic integrity. Copper-dependent redox cycling may add risk of cuproptosis [88,89]. Genetic and epigenetic alterations in redox-regulating pathways further magnify brain vulnerability to these processes [90,91,92,93,94].

#### 4.2.2. Increased Production of ROS by Enzyme’s Dysregulation

##### Nicotinamide Adenine Dinucleotide Phosphate Oxidases

Nicotinamide adenine dinucleotide phosphate (NADPH) oxidases (NOX and DUOX isoforms) are membrane-bound oxidoreductases whose sole function is to generate superoxide anion or hydrogen peroxide by transferring electrons from NADPH to oxygen, fueled by the pentose phosphate pathway. In particular, NOX4 and DUOX1/2 generate hydrogen peroxide [52,95,96].

In the CNS, NOX-derived ROS normally contribute to signaling and defense, but in ASD dysregulation become pathogenic. Neuronal and astrocytic NOX4 activity enhances oxidative distress, while microglial NOX2—consistently upregulated in prefrontal cortex and cerebellum—drives chronic neuroinflammation [53,54]. This hyperactivation destabilizes neuronal and glial membranes via lipid peroxidation, weakens resilience, and promotes ferroptosis or cuproptosis [97,98,99,100]. The resulting alterations compromise synaptic integrity and connectivity, linking enzymatic dysregulation directly to neurodevelopmental deficits [101,102,103].

##### Myeloperoxidase

Myeloperoxidase (MPO) is a heme-containing peroxidase that uses hydrogen peroxide, largely generated by NADPH oxidases, to oxidize chloride into hypochlorous acid, a highly reactive oxidant that preferentially modifies amino groups [104]. Under neuroinflammatory conditions, microglial MPO amplifies oxidative cascades and becomes a key mediator linking oxidative distress with inflammation, thereby shaping neuro-oxi-inflammation [105,106,107,108,109]. Beyond intracellular activity, MPO has been detected in extracellular vesicles, suggesting it can propagate oxidative signals beyond the producing cell. This extracellular dissemination is increasingly associated with ASD and other CNS disorders, bridging local oxidative damage with systemic inflammatory responses [110,111].

##### Nitric Oxide Synthases

Nitric oxide synthases—endothelial (eNOS), neuronal (nNOS), inducible (iNOS), and the putative mitochondrial isoform (mitNOS)—are heme–flavoproteins that convert L-arginine into nitric oxide (NO) and L-citrulline [112]. Their activity requires NADPH, flavins, heme, tetrahydrobiopterin (BH_4_), calmodulin, and stable dimerization [113]. Endothelial NOS and nNOS are constitutive and Ca^2+^/calmodulin-dependent, whereas iNOS is transcriptionally induced during inflammation, producing sustained NO fluxes [112]. Mitochondrial NOS may fine-tune oxidative phosphorylation, but excessive activity inhibits cytochrome c oxidase, reducing ATP and increasing ROS [114,115,116].

As a diffusible messenger, NO regulates cyclic guanyl monophosphate (cGMP) signaling, long-term potentiation and depression, cerebral blood flow, and retrograde synaptic signaling [117]. Yet overproduction in immune or glial cells drives nitrosative stress [118,119]. Under hypoxia or tetrahydrobiopterin deficiency, NOS uncouples, shunting electrons to oxygen and generating superoxide anion [118,119]. Nitric oxide and superoxide anion rapidly combine to form peroxynitrite (ONOO^−^), a potent oxidant that nitrates tyrosine moieties, oxidizes thiols, damages DNA, and disrupts mitochondria [72,76,117,120,121,122]. In ASD, NOS variants, iNOS hyperactivation, and nNOS dysregulation foster chronic redox imbalance, mitochondrial dysfunction, and synaptic disorganization [123]. The convergence of NO excess, peroxynitrite, and ROS crosstalk establishes a pathogenic loop of oxidative–nitrosative stress that critically shapes neurodevelopmental vulnerability [124,125,126].

##### The Unique Potential Role of Gamma-Glutamyl Transferase/Transpeptidase in ASD

**Figure 6:**
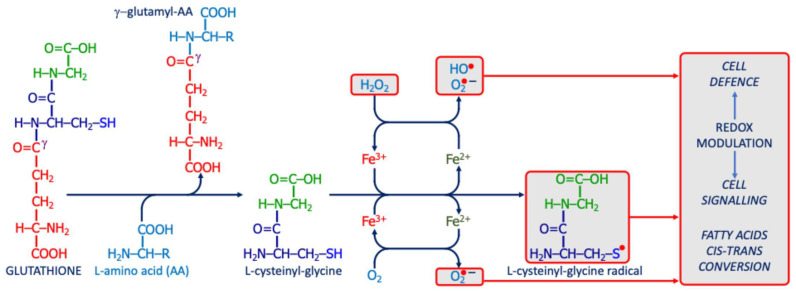
The potential pro-oxidant action of γ-glutamyl-transferase/transpeptidase. The enzyme releases L-cysteinyl-glycine from GSH; this dipeptide can react with iron to generate ROS, producing a radical that modulates redox signaling or disrupts membrane integrity (e.g., via cis–trans isomerization of unsaturated fatty acids).

Gamma-glutamyl transferase/transpeptidase (γ-GT) is a plasma-membrane ectoenzyme that catalyzes the transfer of γ-glutamyl groups from GSH to water or amino acids, sustaining GSH turnover and amino acid transport. When γ-GT is overexpressed or mislocalized, extracellular cysteinyl-glycine can reduce ferric to ferrous iron, fueling Fenton chemistry and hydroxyl-radical formation [127,128]. The resulting thiyl-radical environment promotes cis–trans isomerization of fatty acyl chains and accumulation of trans-fatty acids in neuronal membranes (Figure 6) [129,130]. Such structural changes may reduce membrane fluidity, disrupt lipid rafts, and sensitize lipids to peroxidation, thereby amplifying neuro-oxi-inflammation and increasing ferroptotic risk [131,132]. In the CNS, astrocytic and endothelial γ-GT polymorphisms further modulate this vulnerability [133]. From a translational perspective, γ-GT dysregulation links altered membrane dynamics and synaptic signaling to heightened oxidative burden in ASD [133].

##### Mitochondrial Pro-Oxidant Enzymes

α-ketoglutarate dehydrogenase, succinate dehydrogenase, glycerol-3-phosphate dehydrogenase, aconitase, and dihydroorotate dehydrogenase form a pool of pro-oxidant mitochondrial systems that contribute to ROS production [134,135,136,137,138]. In the CNS, where energy demand and mitochondrial density are high, this production is physiologically intense but must remain tightly regulated [139]. When dysregulated, oxidative overload damages macromolecules and fosters conditions that compromise both neurodevelopment and neurodegeneration [139].

On the outer mitochondrial membrane, MAO-A and MAO-B further add to the burden by degrading dopamine, serotonin, and norepinephrine, generating hydrogen peroxide and ammonia [56]. Genetic polymorphisms, such as MAO-A Variable Number Tandem Repeat (VNTR) variants, influence monoaminergic tone and have been linked to ASD-related neurodevelopmental and behavioral phenotypes [140,141,142]. Monoamine oxidase A predominates in catecholaminergic neurons, whereas MAO-B is enriched in serotonergic neurons and astrocytes, particularly in striatum, substantia nigra, and frontal cortex—regions consistently implicated in ASD. Here, excessive MAO activity or insufficient catalase (CAT) buffering promotes oxidative distress [57,85,143].

From a translational perspective, mitochondrial ROS production and MAO-derived OS directly interfere with neurotransmitter balance, synaptic plasticity, and neuronal survival. This dual impact on energy metabolism and neurotransmission reinforces mitochondria as hubs where biochemical dysregulation translates into ASD neurodevelopmental abnormalities.

##### Other Pro-Oxidant Enzymes

Very long-chain acyl-CoA oxidase (ACOX) initiates peroxisomal β-oxidation by transferring electrons directly to oxygen, generating hydrogen peroxide, the main peroxisomal ROS. Additional oxidases, including D-amino acid oxidase, also contribute. Normally, CAT detoxifies hydrogen peroxide, but when impaired, hydrogen peroxide accumulation drives oxidative distress [144,145,146].

Cyclooxygenases (COX) and lipoxygenases (LOX) oxidize arachidonic acid to prostaglandins and leukotrienes, producing reactive metabolites such as lipid hydroperoxides and 4-hydroxynonenal (4-HNE). Elevated COX-2 and LOX expression in ASD correlate with lipid peroxidation and neuro-oxi-inflammation [58,147]. Fatty-acid desaturases, essential for brain lipid composition, when dysfunctional—often due to Single Nucleotide Polymorphisms (SNPs)—favor ROS production, disrupt lipid metabolism, and contribute to neurodevelopmental deficits [59,148,149].

Cytochrome P_450_ (CYP) enzymes hydroxylate steroids, prostaglandins, leukotrienes, and xenobiotics, generating ROS by-products. Dysregulation alters neurosteroid and eicosanoid balance, enhances oxidative stress, and promotes synaptic dysfunction. Genetic polymorphisms modulate these outcomes [150,151,152,153,154].

Xanthine oxidase (XO), a pathological variant of xanthine dehydrogenase, participates in purine catabolism but shifts to XO during ischemia, reducing oxygen to superoxide and hydrogen peroxide. Upon reperfusion, XO contributes to neuronal oxidative distress in ASD, consistent with elevated serum activity. Xanthine oxidase also reduces nitrites to NO and is partially inhibited by quercetin [155,156,157,158].

The kynurenine pathway has gained prominence. Indoleamine 2,3-dioxygenase and tryptophan 2,3-dioxygenase generate superoxide during tryptophan oxidation, while kynurenine 3-monooxygenase consumes NADPH to form 3-hydroxykynurenine, which auto-oxidizes to radicals and hydrogen peroxide. Quinolinic acid over-activates N-Methyl-D-Aspartate (NMDA) receptors, increasing Ca^2+^-driven ROS and mitochondrial dysfunction [159,160,161,162,163]. In ASD, these alterations amplify neuro-oxi-inflammation and impair neurodevelopment.

## 5. Biological Target Accessibility/Vulnerability Is Impaired in ASD

### 5.1. The Polyunsaturated Fatty Acid Target

**Figure 7:**
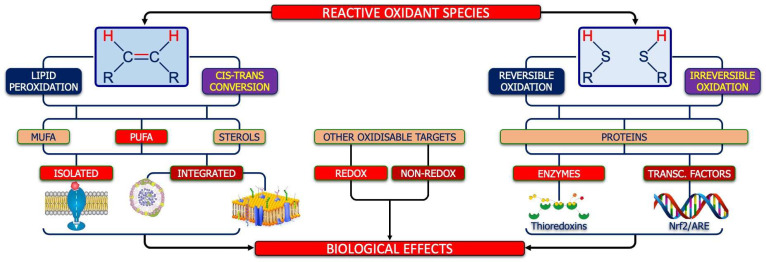
Molecular targets of ROS. As shown on the left side, the first target of ROS is the double bond between carbon atoms, whose oxidation can lead to lipid peroxidation or cis–trans conversion, both affecting monounsaturated fatty acids (MUFA), poly-unsaturated essential fatty acids (PUFA), and sterols. The oxidation of isolated fatty acids (including the PUFA moiety of endocannabinoids) can have an impact on cell reactivity/signaling, while the oxidation of phospholipid-bound fatty acids could have an impact on structure and function of circulating lipo-proteins and/or cell membranes. Globally, lipid peroxidation and/or the isomeric cis–trans conversion can modify the thickness and/or the fluidity of neuronal/glia membranes, with impairment of energetic, metabolic, and signaling flow between the two sides of them. On the other hand, as shown on the right side, ROS can also react, reversibly or irreversibly, with the reduced thiols of cysteine pairs belonging to the same protein (including enzymes, such as thioredoxins, or transcriptional factors such as Nrf2) thus contributing to the adaptative functions of the redox system in nervous system. Finally, as shown in the center of figure, ROS can impact other targets, such as redox-sensitive targets (e.g., oxidants, such as nitric oxide, or antioxidants, such as bilirubin), another adaptative mechanism under the control of the redox system.

Polyunsaturated fatty acids in neuronal and glial membranes are major targets of ROS. In ASD, lipid peroxidation represents a pivotal mechanism linking redox imbalance to neurodevelopmental dysfunction (Figure 7, left side).

Nicotinamide adenine dinucleotide phosphate oxidase hyperactivation is a primary trigger [52]. Increased NOX activity in cerebellum and prefrontal cortex elevates oxidative burden and behavioral abnormalities in BTBR T+ Itpr3tf/J mouse strain (BTBR) mice, while environmental exposures such as phthalates or ethanol intensify these effects [54,164,165]. In ASD children, NOX2 overexpression—partly mediated by toll-like receptor (TLR) 4 signaling—drives neuroinflammation. Crosstalk between NOX and nNOS amplifies ROS/RNS generation, accelerating PUFA oxidation [166].

Peroxidation of PUFA-rich membranes initiates chain reactions producing lipid peroxides. Normally, selenium-dependent glutathione peroxidase (GPx) detoxifies these intermediates, but reduced GPx activity, frequently reported in ASD, allows their accumulation [59,99,100]. Peroxides can react with transition metals, propagating ferroptosis or cuproptosis, or degrade into aldehydes such as malondialdehyde (MDA) and 4-HNE [99,167]. Elevated in ASD plasma and the brain, these aldehydes form adducts with proteins, lipids, and DNA, impairing enzymatic activity and synaptic integrity [59,99,100]. 4-hydroxynonenal also acts as a signaling mediator, activating Nuclear Factor Kappa-Light-Chain-Enhancer of Activated B Cells (NF-κB), Activator Protein-1 (AP-1), and Mitogen-Activated Protein Kinase (MAPK) cascades, reinforcing neuroinflammation [168].

Ferroptosis, an iron-dependent form of regulated cell death, is increasingly implicated in ASD [101,169]. Glutathione peroxidase 4 deficiency and GSH depletion lower the ferroptotic threshold, while mitochondrial dysfunction and iron dysregulation sustain it [102]. Excess ferroptosis during neurodevelopment disrupts pruning and cortical organization [170,171].

Nitric oxide and peroxynitrite also oxidize PUFA, generating nitro-PUFA that, when excessive, compromise membrane fluidity and synaptic signaling [172]. In addition, cis–trans isomerization by thiyl radicals rigidifies membranes, a process worsened by dietary trans-fats [129,173,174]. Such trans-fats may enter via unhealthy diets—whose consumption has been linked to ASD severity—or be produced endogenously under oxidative distress. Lipidomic studies in ASD confirm altered fatty acid composition and increased trans-fat levels [175,176,177].

Altogether, PUFA peroxidation, aldehyde toxicity, ferroptosis, nitro-PUFA accumulation, and trans-fat incorporation converge as key pathogenic drivers, bridging redox imbalance, mitochondrial dysfunction, and neuroinflammation in ASD.

### 5.2. The Thiol Proteins’ Molecular Target

Protein thiol groups are primary targets of reactive oxidants such as hydrogen peroxide and NO [46,178,179,180] (Figure 7, right side). In ASD, mitochondrial dysfunction, NADPH oxidase hyperactivation, and chronic neuroinflammation increase ROS, amplifying thiol oxidation and nitrosylation with major effects on neuronal physiology [54,164,181,182,183].

Under physiological conditions, NO from neuronal and inducible NOS regulates neurogenesis and plasticity through cGMP signaling and controlled S-nitrosylation [55,112]. In ASD, persistent iNOS over-activation drives nitrosative stress and peroxynitrite formation; elevated iNOS and nitrosative lesions in Purkinje cells highlight cerebellar vulnerability [118,120,123].

Hydrogen peroxide-mediated thiol oxidation (sulfenylation, disulfides, glutathionylation, and overoxidation) alters enzyme activity and protein interactions [178,179,180]. Redox-sensitive proteins—including glutamate receptors, ion channels, tubulin, and actin—malfunction, leading to impaired neurotransmission, cytoskeletal collapse, and synaptic disorganization; severe oxidation may trigger disulfidoptosis, a thiol-dependent cell death linked to mitochondrial dysfunction [184,185,186].

Aberrant nitrosylation adds disruption: NMDA subunits and scaffolds undergo excessive S-nitrosylation, disturbing the excitatory/inhibitory balance [187]. In SH3 and Multiple Ankyrin Repeat Domains 3 (SHANK3)-deficient mice, widespread hypernitrosylation correlates with defective trafficking, impaired glutamatergic signaling, and ASD-like behaviors [95,188]. Peroxynitrite irreversibly nitrates tyrosines (3-nitrotyrosine), a consistent ASD marker, while NO-mediated modification of mitochondrial proteins undermines oxidative phosphorylation [72].

Nitrosative stress also reprograms transcription by oxidizing Kelch-like ECH-associated protein 1 (KEAP1), aberrantly activating Nuclear Factor erythroid 2–related Factor 2 (Nrf2), and nitrosylating mechanistic target of rapamycin (mTOR) or glyceraldehyde-3-phosphate dehydrogenase (GAPDH) [189,190]. These changes promote apoptosis, impair neurogenesis, and affect vascular regulation; systemically, thiol modifications compromise differentiation, axonal guidance, and synaptic stability, mirrored by elevated plasma nitrotyrosine/nitrates and reduced thiols [72,117,125]. Tetrahydrobiopterin deficiency, by uncoupling NOS, further distorts NO metabolism [113,191].

In summary, chronic nitrosative stress integrates oxidative distress, excitotoxicity, and maladaptive plasticity, consolidating thiol oxidation, nitrosylation, and disulfidoptosis as key mechanisms linking redox imbalance to abnormal neurodevelopment in ASD [124,125,126].

### 5.3. Other Molecular Targets

Protein carbonylation—irreversible ROS-driven addition of carbonyl groups—causes loss of function and resistance to proteasomal degradation, promoting accumulation and damage. Elevated carbonyls in the plasma and brain of ASD patients support their dual value as biomarkers and effectors [192].

Mitochondrial DNA (mtDNA), lacking histones and with limited repair, is highly vulnerable: superoxide and hydroxyl radicals induce 8-oxo-2′-deoxyguanosine, strand breaks, and deletions that impair oxidative phosphorylation and further raise ROS [193,194,195]. Accumulated mtDNA damage in frontal cortex and cerebellum underscores mitochondrial fragility in ASD [193,194,195].

At the nuclear level, oxidative stress perturbs Cytosine-phosphate-Guanine (CpG) methylation and histone marks, altering programs crucial for synaptogenesis, neurogenesis, and immune regulation [196,197]. These changes link oxidative injury to altered development and immune dysregulation in ASD [196,197].

Translationally, the convergence of protein carbonylation, mtDNA injury, and epigenetic shifts shows how oxidative stress reshapes cellular integrity and gene control, disturbing neurodevelopmental timing and immune homeostasis within ASD’s multifactorial pathology [12,62,63,64,65].

### 5.4. Redox-Sensitive Molecular Pathways’ Targets

In ASD, whole metabolic/signaling pathways are reprogrammed by redox imbalance [12,48,76]. Central is the thiol pathway: thiol–disulfide exchanges govern redox sensing [15,46,47]. A consistent hallmark is reduced plasma thiols with increased disulfides, indicating a systemic oxidative shift that compromises synaptogenesis, plasticity, and neuroimmune regulation; aberrant disulfide bonding favors misfolding, axonal impairment, and disulfidoptosis-driven vulnerability [184,185,186,198].

One-carbon metabolism, essential for methyl transfer and epigenetic control, is also impaired: reduced folate, a lower S-adenosylmethionine/S-adenosylhomocysteine (SAM/SAH) ratio, hyperhomocysteinemia, and global hypomethylation affect differentiation, plasticity, and neurotransmission [199,200]. Methylenetetrahydrofolate reductase (MTHFR) variants aggravate these effects, especially with folate deficiency. Metabolomics further links methylation defects with oxidative biomarkers, reinforcing reciprocal redox–epigenetic interactions [199,200,201,202].

The endocannabinoid system—anandamide, 2-arachidonoylglycerol (2-AG), cannabinoid (CB) 1/2 receptors, and enzymes—is redox-sensitive and modulates excitatory/inhibitory balance through retrograde control of glutamate and gamma-aminobutyric acid (GABA) release [203,204]. In ASD, oxidative distress reduces arachidonic acid pools, alters receptor expression, and impairs MAPK and PI3K (Phosphoinositide 3-kinase) Protein kinase B (PI3K–Akt) signaling; clinically, decreased anandamide and CB1 correlate with social deficits and oxidative imbalance [205,206,207].

Transcriptional programs are compromised: NRF2 is underexpressed, limiting induction of heme oxygenase-1 (HO-1), γ-glutamyl-cysteine ligase (GGL), and GPx, thereby undermining GSH homeostasis, lowering the ferroptotic threshold, and sustaining neuroinflammation [48,103,190,208,209,210,211,212]. Peroxisome proliferator-activated receptor-γ coactivator-1α (PGC-1α), crucial for mitochondrial biogenesis, is impaired by epigenetic changes and mtDNA damage, leading to energy deficits and heightened oxidative sensitivity [213,214,215,216].

Gasotransmitters add fragility. Reduced carbon monoxide (CO) impairs cGMP signaling and associates with severity/autoimmunity [209,217]. Carbon dioxide dysregulation perturbs pH, excitability, and neurovascular coupling [218,219]. Excess hydrogen sulfide (H_2_S) can induce oxidative damage and mitochondrial dysfunction [220,221].

Overall, ASD reflects systemic reprogramming of thiol biology, one-carbon metabolism, endocannabinoid tone, transcriptional networks, and gasotransmitter signaling—converging on impaired bioenergetics, maladaptive plasticity, and aberrant epigenetic programming.

## 6. Decreased Bioavailability/Activity of Reducing/Antioxidant Systems in ASD

### 6.1. The Neglected Role of Glucose-6-P-Dehydrogenase

Redox dysfunction in ASD is mirrored by altered antioxidant levels/activities across biofluids and tissues [71,222,223,224]. Central is glucose-6-phosphate dehydrogenase (G6PD), which generates NADPH via the pentose phosphate pathway—the main reservoir of cellular reducing power [225]. Nicotinamide adenine dinucleotide phosphate sustains reductive biosynthesis (cholesterol, membrane fatty acids), fuels NADPH oxidases, and recycles oxidized components such as GSH [226].

Glucose-6-phosphate dehydrogenase deficiency, reported in ASD, reduces NADPH and disrupts the redox network, fostering neuro-oxi-inflammatory cascades [227,228,229]. Experimental data show that low NADPH impairs synaptogenesis and alters folate metabolism, with adverse effects on myelination and synaptic performance [225].

Translationally, G6PD deficiency represents a pivotal metabolic vulnerability: by weakening systemic antioxidant defenses, it links cellular redox imbalance to disrupted maturation and connectivity, contributing to the neurodevelopmental phenotype of ASD [225,227].

### 6.2. Glutathione Pathway

In ASD, redox dysfunction is closely tied to impaired GSH metabolism [230]. As the most abundant low-molecular-weight thiol, GSH is vital in the CNS, where high oxidative metabolism constantly challenges redox balance. Beyond its antioxidant role, it regulates detoxification, thiol homeostasis, signaling, and epigenetic programming [11,13,170].

Evidence shows systemic GSH depletion in plasma, lymphocytes, and brain regions such as cerebellum, prefrontal cortex, and hippocampus [231,232,233]. Environmental toxicants like mercury and lead aggravate this decline, particularly with low sulfur amino acid intake [234,235]. Glutathione biosynthesis depends on GCL, under NRF2 control [236]. In ASD, attenuated NRF2 responses reduce GCL and GPx expression, forming a dysfunctional Nrf2–GSH loop [236,237].

Polymorphisms in GPX1 and glutathione-S-transferases (GSTM1, GSTT1, GSTP1) further weaken defenses [94,238]. This sustains lipid peroxidation and lowers the ratio between reduced and oxidized glutathione (GSH/GSSG ratio)—a robust ASD biomarker —correlating with impaired energy metabolism, synaptic plasticity, and epigenetic regulation [99,231,232].

Functionally, reduced GSH lowers GPx activity, enabling lipid peroxide accumulation, ferroptotic vulnerability, and reactive aldehyde formation (4-HNE, MDA), which form covalent adducts with macromolecules and amplify microglial inflammation [59,99,100]. Chronic dysregulation perpetuates oxidative distress, DNA damage, and abnormal neurodevelopment [19,239,240].

Altered GSH metabolism also reduces the SAM/SAH ratio, impairing DNA and histone methylation [241,242], undermining epigenetic control of neuronal differentiation and immune regulation [243,244]. Normally, glutathionylation protects proteins and tunes signaling; in ASD, impaired glutathionylation destabilizes protein function and mitochondrial bioenergetics [243,244].

Within the system, GPx1 is ubiquitous, while GPx4 uniquely reduces lipid hydroperoxides and prevents ferroptosis [245,246]. Glutathione peroxidase 4 deficiency, increasingly linked to ASD, heightens neuronal vulnerability, with variants such as GPX1 Pro198Leu reinforcing risk [230,234].

Astrocyte–neuron coupling adds another dimension: astrocytes synthesize and export GSH for neuronal uptake, normally enhanced by norepinephrine, but disrupted in ASD [247].

Altogether, impaired synthesis, excessive consumption, defective recycling, genetic susceptibility, and disrupted glia–neuron cooperation converge on persistent redox imbalance. This promotes lipid peroxidation, thiol oxidation, ferroptosis, and abnormal protein modifications, culminating in synaptic dysfunction. By impairing pruning, dendritic maturation, and epigenetic regulation, GSH dysfunction emerges as a defining feature of redoxomic dysregulation in ASD [12,48,62,63,64,76,248].

### 6.3. Other Relevant Antioxidant Enzymes

Catalase, a peroxisomal enzyme, decomposes hydrogen peroxide into water and oxygen, preventing oxidative “friendly fire”. Abundant in astrocytes and neurons, it helps maintain redox balance [249,250,251]. Autism Spectrum Disorder studies report inconsistent findings [75]: reduced activity in post-mortem brain tissue versus compensatory increases in others [158,252]. These discrepancies highlight the limitations of isolated biomarkers and the need for integrated redoxomic evaluation [230]. Heme oxygenase-1, another NRF2-inducible enzyme, degrades heme into biliverdin, CO, and iron. Biliverdin is reduced to bilirubin (antioxidant), CO acts as a cytoprotective signal, and free iron induces ferritin. In ASD, serum HO-1 is reduced while KEAP1 and Nrf2 are elevated, suggesting OS with inadequate HO-1 response [48,217,253].

8-Oxoguanine DNA glycosylase-1 (OGG1) repairs mutagenic 8-oxoguanine lesions. Neuronal OGG1 protects genomic stability, but its deficiency leads to lesion accumulation, a condition linked to ASD [254,255,256].

Paraoxonase-1 (PON1), an HDL-bound enzyme, hydrolyzes organophosphates and lipid hydroperoxides [257,258]. Polymorphisms (Q192R, L55M) modulate activity and ASD risk [259]. Reduced PON1 activity, sometimes uncoupled from oxidative markers, interacts with gene–environment factors such as prenatal pesticide exposure [257,260].

Translationally, these enzymes illustrate how deficits in detoxification, DNA repair, and antioxidant buffering converge to exacerbate oxidative distress in ASD, with genetic and environmental interactions shaping vulnerability and outcomes.

## 7. The Controversial Role of Superoxide Dismutases in ASD

Superoxide dismutases are central redox enzymes with three isoforms: SOD1 (Cu/Zn-SOD) in cytoplasm and intermembrane space, SOD2 (Mn-SOD) in mitochondria, and SOD3 (EC-SOD) extracellularly. All convert superoxide to oxygen and hydrogen peroxide. Hydrogen peroxide, however, if not neutralized, may promote a condition of oxidative distress, despite the protective action of SOD against the superoxide anion [261].

This duality makes SOD a “biochemical paradox”: enzymes considered antioxidant, yet potentially pro-oxidant. In Down syndrome, trisomy 21-driven SOD1 overexpression increases hydrogen peroxide production, aggravating stress [262].

In ASD, results are inconsistent. Most studies report reduced SOD activity in blood or immune cells, correlating with elevated oxidative biomarkers and more severe phenotypes [261,263,264,265]. A low SOD/CAT ratio reflects poor enzymatic cooperation, favoring lipid and DNA oxidation [252,266]. Conversely, some describe increased SOD activity, which, without efficient detoxification, paradoxically intensifies oxidative distress [267,268]. Variability likely reflects genetic polymorphisms, tissue specificity, and comorbidities [71,269,270].

Overall, in ASD, SOD act as a “double-edged sword”: essential for superoxide clearance but pathogenic when hydrogen peroxide removal is inadequate. Translationally, this paradox is critical: SOD and SOD-mimetics are often proposed as antioxidants, yet their efficacy may be limited or harmful without adequate downstream detoxification. In ASD, where redox balance is fragile, such interventions must be approached with caution [158,191,271,272].

## 8. Impairment of Neurons/Glia Subcellular Compartments in ASD

Redox dysfunction in ASD does not remain an abstract imbalance but materializes within specific subcellular compartments. At the membrane, lipid peroxidation and trans-fat incorporation compromise fluidity and receptor organization. In the cytoplasm and mitochondria, ferroptotic activation and impaired antioxidant buffering undermine bioenergetics, neurotransmission, and protein stability. In the nucleus, oxidative and nitrosative stress damage DNA and reprogram epigenetic landscapes, reshaping developmental trajectories. These effects extend beyond neurons: glial cells, particularly microglia, undergo maladaptive activation and chronic neuroinflammation. Such compartment-specific alterations represent the neurobiological foundation of ASD and are detailed in the following sections [6,7,35,36,48,51,65,71,73,74,239,273,274].

### 8.1. Mitochondrial Dysfunction and Altered Microbiota–Gut–Brain–Mitochondria Axis

Mitochondrial dysfunction in ASD is often simplified as reduced ATP synthesis with ROS overproduction. While relevant, this view overlooks mitochondria’s broader functions in apoptosis, neurotransmitter turnover, redox signaling, and immune control. Alterations here profoundly affect brain development and connectivity, making mitochondria key hubs of ASD pathophysiology [181,274,275,276].

In ASD, impairment extends beyond oxidative phosphorylation to global metabolic and structural abnormalities. Reduced ATP and ROS excess destabilize the Krebs cycle, β-oxidation, and urea cycle, leading to excitotoxicity and synaptic failure. Disturbed polyamine metabolism and steroidogenesis impair chromatin remodeling, neurosteroid synthesis, and excitatory/inhibitory balance [274,275,276,277]. At the outer membrane, MAO activity exacerbates oxidative stress through hydrogen peroxide and aldehydes, altering dopamine and serotonin turnover [181,278,279].

Persistent oxidative injury, coupled with defective mitophagy, favors dysfunctional organelles, apoptosis, and senescence [278,279,280]. Genetic and epigenetic variants in respiratory chain or redox regulation intensify vulnerability, especially in high-demand areas such as the prefrontal cortex, hippocampus, and cerebellum during early development [181,278].

This fragility is reinforced by the evolutionary reciprocity between mitochondria and gut microbes [281,282]. In ASD, dysbiosis reduces butyrate—an anti-inflammatory PGC-1α activator—while increasing propionate, which impairs respiration, raises ROS, and induces ASD-like behaviors [283,284]. Increased intestinal permeability allows lipopolysaccharides and sulfur metabolites into circulation, sustaining systemic inflammation and oxidative distress [285]. Conversely, mitochondrial dysfunction in enterocytes and neurons undermines colonization by symbiotic microbes, destabilizing holobiont equilibrium [215,286,287].

Clinical evidence shows reduced *Lactobacillus* and *Bifidobacterium* and increased *Clostridia* in ASD [288]. Some *Lactobacillus* strains enhance NRF2-dependent antioxidant defenses, exemplifying host–microbe redox dialogue [236,289]. Polyphenols such as curcumin remodel microbiota and generate metabolites that cross the blood–brain barrier, modulating mitochondria, inflammation, and epigenetic programming [290,291].

Thus, mitochondrial dysfunction and dysbiosis represent interconnected consequences of redox imbalance in ASD. Rooted in evolutionary symbiosis, their disruption links genetic susceptibility, environment, and microbial ecology to impaired neurodevelopment, positioning mitochondria and microbiota as central amplifiers of oxidative distress [283,284,292].

### 8.2. Redox-Mediated Cell Membrane Dysfunction in ASD

#### 8.2.1. Abnormalities in Neurons: Neurotransmission, Plasticity and Synaptic Functions, Morphogenesis

In ASD, redox dysfunction destabilizes neuronal membranes and synaptic machinery, linking oxidative distress to cognitive and behavioral deficits. Neuronal membranes, rich in PUFA and thiol proteins, are highly vulnerable to oxidative/nitrosative stress. Lipid peroxidation reduces fluidity, disrupts receptor clustering, and alters ion channel conductance, while protein oxidation disturbs excitatory/inhibitory balance [72,293,294].

Dopaminergic pathways are particularly sensitive: dopamine auto-oxidation generates quinones, while MAO produces hydrogen peroxide, damaging membranes and neurotransmitter turnover [85,143]. Tryptophan metabolism is redirected by indoleamine 2,3-dioxygenase toward quinolinic acid, a neurotoxic NMDA agonist, while reducing kynurenic acid, enhancing excitotoxicity [295,296]. Gamma-amino-butyric-ergic tone weakens as glutamate decarboxylase (GAD)65/67 undergoes nitrosylation and downregulation, especially in the cerebellum and parietal cortex [293,294]. Exceeding NO, normally needed for plasticity and oxytocin signaling, instead drives aberrant S-nitrosylation, undermining oxytocin–GABA interactions crucial for social cognition [72].

Synaptic proteins such as Soluble N-ethylmaleimide-Sensitive Factor Attachment Protein Receptor (SNARE) complexes, neuroligins, neurexins, and Shank3 are oxidatively modified, impairing vesicle trafficking and receptor anchoring [12,20,51,297]. Mitochondrial dysfunction compounds these effects by limiting ATP supply and calcium buffering, while microglial hyperactivation replaces physiological pruning with neuro-oxi-inflammation [23,51].

Neuroplasticity is severely impaired. Reduced Brain-Derived Neurotrophic Factor (BDNF) limits dendritic arborization, while oxidative modifications of NMDA receptors compromise long-term depression/long-term potentiation (LTP/LTD). Cytoskeletal destabilization further weakens dendritic dynamics [239,298,299]. Glutathione depletion correlates with oxidative injury in the hippocampus, cerebellum, and temporal cortex, reinforcing excitatory/inhibitory imbalance [239,300].

Clinically, these molecular changes manifest as immature dendritic arborization, defective axonal guidance, inefficient pruning, and aberrant connectivity [299]. Social deficits reflect oxytocin–GABA dysfunction, language impairments stem from temporal–frontal disconnection, and sensory hypersensitivity arises from maladaptive excitatory/inhibitory imbalance [298,299].

Overall, redox-driven membrane fragility, neurotransmitter imbalance, protein oxidation, and impaired plasticity converge into a cascade that reshapes neural circuits into hyperconnected yet inefficient networks, providing a biochemical substrate for ASD phenotypes [299].

#### 8.2.2. Abnormalities in Microglia: Neuro-Oxi-Inflammation

In ASD, neuro-oxi-inflammation arises from the convergence of OS and inflammation into a self-sustaining loop that derails neurodevelopment [300]. Central drivers include NOX enzymes, NOS isoforms, and γ-GT, which generate ROS, NO, and cysteinyl-glycine, fueling Fenton chemistry. These reactions turn membranes into pro-oxidant platforms that sustain maladaptive inflammation [98,120,121,301,302].

Although intertwined, OS and inflammation differ mechanistically: OS is a rapid electron-transfer response producing hydrogen peroxide as a signal, whereas inflammation relies on cytokines and transcriptional programs, slower and more energy-consuming. Oxidative distress can exist without inflammation, but rarely vice versa, underscoring redox imbalance as the primary neural trigger [110,303,304,305].

Maternal immune activation exemplifies this link. Infections or autoimmunity during pregnancy raise ASD risk, as shown in polyinosinic:polycytidylic acid (poly I:C) and lipopolysaccharide (LPS) models [300,306]. Maternal cytokines, such as 6 and 17A interleukins, and tumor necrosis factor (TNF)-α cross the placenta and immature blood–brain barrier, pushing fetal microglia into pro-inflammatory states [182,307]. These cells, evolutionarily related to macrophages, release cytokines and ROS, impairing pruning and maturation Disruption of C-X3-C motif chemokine ligand 1 (fractalkine)—C-X3-C motif chemokine receptor 1 (CX3CL1–CX3CR1) and Cluster of Differentiation 200—CD200 receptor (CD200–CD200R) signaling destabilizes neuron–microglia crosstalk, perpetuating hyperactivation [308].

Myeloperoxidase further amplifies oxidative cascades, while mitochondrial dysfunction sustains the cycle via ROS overproduction and impaired energy buffering. Post-mortem studies confirm reduced complex activity and oxidative injury [105,107]. Epigenetic reprogramming of microglia by maternal cytokines consolidates these predispositions [309,310].

Ultimately, oxidative distress, inflammation, and mitochondrial dysfunction converge to impair pruning, plasticity, and connectivity. The result is dysconnectivity with local synaptic overgrowth, a hallmark of ASD. Clinically, these processes manifest as deficits in language, social cognition, and sensory integration. From an evolutionary perspective, neuro-oxi-inflammation reflects the maladaptive persistence of an ancient defense system, chronically activated during critical windows and predisposing to later neuro-oxi-inflammaging [311,312,313].

### 8.3. Redox-Mediated Lysosome Dysfunction in ASD

Lysosomal dysfunction represents another outcome of redox imbalance, linking oxidative stress, defective clearance, and premature aging. Normally, lysosomes sustain homeostasis through autophagy and mitophagy, eliminating damaged mitochondria [314,315]. In ASD, autophagic flux is impaired, while dysregulated mTOR and PI3K–Akt signaling suppress clearance [316,317,318]. This failure promotes neuronal and glial senescence; senescent microglia release cytokines and proteases that impair pruning, while neural precursor senescence limits neurogenesis and myelination [316,317,318]. Clinically, these processes manifest as cortical dysconnectivity and altered dendritic spine density [319,320,321].

### 8.4. Redox-Mediated Peroxisomal Dysfunction in ASD

Peroxisomes are central redox regulators. They oxidize very long-chain fatty acids and neutralize hydrogen peroxide via CAT. When detoxification is impaired—by genetic defects or CAT insufficiency—hydrogen peroxide accumulates, generating hydroxyl radicals that drive lipid peroxidation and irreversible injury [322]. In the developing brain, this undermines membrane integrity, plasmalogen metabolism, and white matter connectivity [323]. Evidence in ASD includes abnormal CAT activity, altered lipid profiles, and increased peroxidation [239]. Thus, peroxisomes emerge as metabolic hubs converging with mitochondrial dysfunction and inflammation [239].

### 8.5. Redox-Mediated Endoplasmic Reticulum Dysfunction in ASD

The endoplasmic reticulum (ER) integrates redox balance, lipid metabolism, and protein folding. Oxidative distress destabilizes these processes, altering membrane composition, receptor activity, and steroid synthesis, thereby fueling neuroinflammation and excitatory/inhibitory imbalance. Misfolded proteins accumulate, triggering ER stress and the unfolded protein response (UPR). Initially adaptive, chronic UPR drives pro-apoptotic, pro-inflammatory, and pro-oxidant cascades. In ASD, sustained ER stress has been observed in the temporal cortex and cerebellum, impairing calcium regulation, mitochondrial function, and neuroimmune signaling, thereby disrupting synaptic maturation [159,324,325].

### 8.6. Redox-Mediated Cytoskeleton Dysfunction in ASD

Cytoskeletal integrity is highly sensitive to OS as reported in some biological models. Oxidation of tubulin cysteines destabilizes microtubule polymerization, impairing axonal guidance and dendritic arborization [326]. Actin filaments and the acto-myosin system are equally vulnerable: ROS/RNS disrupt filament turnover and growth cone motility, hindering neurite extension [327]. At synapses, oxidative modifications of scaffolding proteins compromise dendritic spine density and morphology, consistently observed in ASD [328]. Redox interference with RAT-Sarcoma (RAS) signaling further disrupts LTP and pruning [329]. Mitochondrial and ER dysfunction exacerbate these effects by limiting ATP and calcium buffering [330]. The outcome is defective neuronal migration, abnormal synaptic maturation, and reduced plasticity, translating molecular stress into disorganized connectivity [331].

### 8.7. Redox-Mediated Nuclear Dysfunction in ASD

The nucleus is a final target of redox imbalance. Oxidative/nitrosative distress triggers guanine oxidation to 8-hydroxy-2′-deoxyguanosine, impairing replication and transcription [332,333]. In parallel, epigenetic marks are altered: DNA methylation, histone acetylation, and microRNA profiles shift, reshaping gene expression [67]. Redox-sensitive transcription factors such as Nrf2, NF-κB, and hypoxia inducible-factor (HIF) 1α become persistently dysregulated, sustaining pro-oxidant and inflammatory programs [48,316,334]. Enzymes like DNA methyltransferases and histone deacetylases, dependent on reducing equivalents, are impaired, amplifying epigenetic instability [66,68]. In ASD, abnormal methylation of synaptic and neurodevelopmental genes correlates with clinical severity, stabilizing oxidative insults into pathogenic trajectories. Thus, nuclear dysfunction anchors redox imbalance to persistent neurodevelopmental reprogramming, consolidating oxidative stress as a central mechanism of ASD [335,336,337].

## 9. Conclusions and Future Perspectives

The pathogenesis of ASD can be conceptualized as a sequence of reverberating waves originating from dysfunction of the redox system.

**Figure 8:**
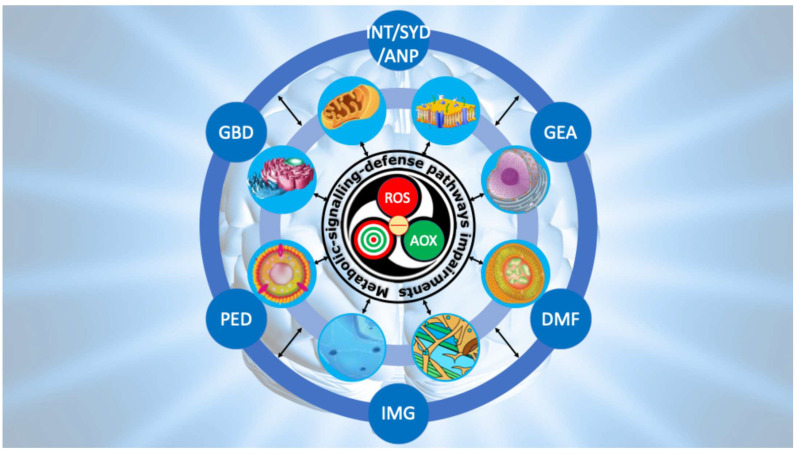
Ripple model to conceptualize the progression from primary redox dysfunction to the clinical phenotype of ASD. The model depicts three interconnected waves linking primary redox dysfunction to the clinical phenotype of ASD. The first wave arises from redox imbalance, which triggers a self-sustaining derailment of metabolic, signaling, and defense pathways. The second wave reflects the progressive collapse of neuronal and glial compartments, extending from the cell membrane to the nucleus. The third wave represents the ultimate neurodevelopmental outcome, characterized by impaired neurotransmission (INT), synaptic dysfunction (SYD), abnormal neuronal plasticity (ANP), genetic and epigenetic alterations (GEA), defective mitophagy (DMF), impaired morphogenesis (IMG), peroxisomal disorders (PED), and gut–brain axis disruption (GBD). Note: the calcium role is not included.

As depicted in Figure 8, the first wave corresponds to the primary collapse of the redox network, where the physiological equilibrium among oxidants, redox-sensitive targets, and reducing agents is lost. What is normally adaptive redox signaling becomes chronic oxidative distress.

The second wave reflects propagation of this imbalance into redox-dependent pathways, including mitochondrial bioenergetics, neurotransmitter turnover, GSH homeostasis, lipid metabolism, and unfolded protein responses, all critically impaired in ASD.

The third wave encompasses cellular and subcellular consequences: mitochondrial inefficiency, impaired gut–brain dialogue, peroxidized neuronal membranes, defective neurotransmission, maladaptive microglial activation fueling neuro-oxi-inflammation, lysosomal MPO-driven cascades with defective mitophagy and premature senescence, peroxisomal insufficiency with unchecked lipid peroxidation, ER stress, cytoskeletal instability, and nuclear genetic–epigenetic reprogramming.

From this third wave emerges the decisive link to neurodevelopmental deviation. When the compartments sustaining synaptic formation, pruning, and plasticity are persistently altered, the developmental trajectory of the brain is redirected. Aberrant connectivity, abnormal spine morphology, disrupted circuit refinement, and faulty integration of sensory, motor, and cognitive networks crystallize into the clinical phenotype of autism. In this sense, the reverberating model explains how an initial molecular imbalance can, through successive layers of perturbation, culminate in the complex manifestations of ASD.

The novelty of this work lies not only in integrating redox dysfunction across multiple biological levels into a unified pathogenetic framework, but also in embedding it in an ecological–evolutionary perspective. Rooting the model in the dual symbiosis that defines eukaryotic life—the ancient endosymbiosis of mitochondria with ancestral bacteria and the dynamic symbiosis with the gut microbiota—frames autism within the broader concept of One Health. Neurodevelopment thus emerges not solely from human genetics or brain-intrinsic processes, but from a continuous biochemical dialogue with microbial partners. Perturbation of these ancient redox-based symbioses reverberates from molecular imbalance to systemic developmental deviation.

Compared with previous literature, this model represents a conceptual advancement. Earlier studies emphasized isolated mechanisms—mitochondrial dysfunction, GSH depletion, neuroinflammation—without fully connecting them within the dynamics of the redox system. By adopting a redoxomic systems biology perspective, we trace the reverberations of imbalance through metabolic pathways, organelle dysfunction, and nuclear reprogramming, bridging molecular pathology with clinical phenotype.

Nonetheless, the model has limitations. It derives from heterogeneous, often cross-sectional data and does not fully capture the temporal sequence from prenatal life to childhood. Bidirectional interactions—between OS and inflammation, or mitochondrial dysfunction and synaptic failure—blur cause and consequence. Patient variability, shaped by genetics, environment, and comorbidities, further challenges universality.

Despite these caveats, the reverberating wave model highlights promising avenues. On the diagnostic side, redox-based biomarkers—enzymatic activities (NADPH oxidases, MAO, MPO), redox couples (GSH/GSSG), lipid peroxidation products, and advanced protein oxidation derivatives—offer dynamic insight into redox performance. Integrated panels, aligned with redoxomic principles, may enable patient stratification, early identification, and real-time monitoring [338]. Beyond classical biomarkers, innovative strategies such as redox imaging, extracellular vesicle analysis, microbiota-derived signals, and genetic/epigenetic redox profiles may expand precision diagnostics [7,121,265,339].

On the therapeutic side, nutraceuticals represent potential tools to modulate redox homeostasis in a personalized way. Bioactive compounds—polyphenols, omega-3 fatty acids, sulfur metabolites, and redox-active vitamins—exert antioxidant effects while influencing signaling, epigenetics, and microbiota composition. Equally promising are novel approaches, including (i) modulation of bitter taste receptors (TAS2Rs) [340], which regulate NO and Ca^2+^ pathways; (ii) control of microbiota-derived redox modulators [341]; and (iii) photobiomodulation [342], which targets mitochondrial chromophores to re-establish redox balance and attenuate microglial activation. These interventions should be viewed not as generic adjuncts but as specific modulators of redox circuits, capable of restoring physiological eustress during critical windows of neurodevelopment.

In conclusion, the reverberating wave model underscores that ASD is best understood as the progressive unfolding of redox system dysfunction across metabolic, cellular, and structural domains, ultimately reshaping neurodevelopment. Embedded within the ecological–evolutionary framework of One Health, autism reflects not only molecular vulnerability but also the fragility of symbiotic networks sustaining human life. While further longitudinal studies are required, this framework coherently links molecular imbalance to clinical phenotype and supports exploration of redox-based biomarkers, nutraceutical strategies, and theranostic innovations as future pillars of precision medicine in ASD.

## Figures and Tables

**Table 1:** Classification of reactive species involved in redox system.

Chemical Species	Formula	Class	Chemical Species	Formula	Class
Singlet oxygen	^1^O_2_	Non radical	Nitric oxide	NO^•^	Radical
Superoxide anion (Reducing species)	O_2_^•−^	Radical	Nitrous acid	HNO_2_	Non radical
Ozone	O_3_	Non radical	Nitric tetroxide	N_2_O_4_	Non radical
Hydroxyl radical	HO^•^	Radical	Nitric trioxide	N_2_O_3_	Non radical
Hydrogen peroxide	H_2_O_2_	Non radical	Peroxynitrite	ONOO^−^	Non radical
Alkyl radical	R^•^	Radical	Peroxynitrous acid	ONOOH	Non radical
(Alkyl-)peroxyl radical	ROO^•^	Radical	Nitronium cation	NO_2_^+^	Non radical
(Alkyl)hydroperoxide	ROOH	Non radical	(Alkyl)peroxynitrite	ROONO^−^	Non radical
Semiquinone (from CoQ10)	Q^•^	Radical	Hypochlorous acid	HClO	Non radical
Tocopheryl (from vit-E)	E-O^•^	Radical	Thiyl	-S^•^	Radical

**Table 2:** Chemical structure, main sources of production, mean migration distance, half-life (T_1/2_), main molecular targets, and scavenging systems of the most common oxygen-centered reactive oxygen species.

Reactive Oxygen Species	Main Sources of Production	Mean Migration Distance	T_1/2_	Main Molecular Targets	Scavenging Systems
Singlet oxygen 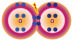	Plasmamembrane Mitochondria	30 nm	1–4 μs	PUFA double bonds Protein coupled thiol	Carotenoids Tocopherols
Superoxide anion 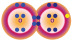	Plasmamembrane Mitochondria	30 nm	1–4 μs	Iron-Sulphur clusters	Superoxide dismutases
Hydroxyl radical 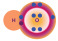	Membranes Mitochondria Cytosol	1 nm	1 μs	Any organic molecule (rapidly reacting with DNA)	Flavonoids
Hydrogen peroxide 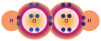	Plasmamembrane Mitochondria Peroxisomes	1 μm	1 ms	Preferably cysteine’s moieties	Catalase Glutathione peroxidases

## Data Availability

No new data were created or analyzed in this study. Data sharing is not applicable to this article.

## Abbreviations

The following abbreviations are used in this manuscript:

2-AG	2-Arachidonoylglycerol
4-HNE	4-Hydroxynonenal
ACOX	Very-long-chain acyl-CoA oxidase
AOX	Reducing/Antioxidant (species)
AP-1	Activator Protein 1
ASD	Autism Spectrum Disorder
BDNF	Brain-Derived Neurotrophic Factor
BTBR	BTBR T+ Itpr3tf/J mouse strain (ASD model)
CAT	Catalase
CB	Cannabinoid (receptors)
CD200–CD200R	Cluster of Differentiation 200-Cluster of Differentiation 200 Receptor
cGMP	Cyclic Guanosine Monophosphate
CNS	Central Nervous System
CO	Carbon monoxide
COX	Cycloxygenase
CpG	Cytosine-phosphate-Guanine
CX3CL1–CX3CR1	Fractalkine—chemokine receptor 1
CYP	Cytochrome P_450_
DUOX	Dual Oxidase (NADPH oxidase isoform)
ER	Endoplasmic Reticulum
G6PD	Glucose-6-phosphate dehydrogenase
GABA	Gamma-aminobutyric acid
GAD (GAD65/67)	Glutamate Decarboxylase (65/67)
GAPDH	Glyceraldehyde-3-phosphate dehydrogenase
γ-GT	γ-glutamyltransferase/transpeptidase
GPx	Glutathione Peroxidase
GSH	Reduced glutathione/glutathione monomer
GSSG	Oxidized glutathione/glutathione dimer
GSTM	Glutathione S-Transferase Mu class
GSTP	Glutathione S-Transferase Pi class
GSTT	Glutathione S-Transferase Theta class
HIF-1α	Hypoxia-Inducible Factor 1-Alpha
HO-1	Heme Oxygenase 1
KEAP1	Kelch-like ECH-associated protein 1
LOX	Lipoxygenase
LPS	Lipopolysaccharide
LTD	long-term depression
LTP	long-term potentiation
MAO	Monoamine Oxidase
MAPK	Mitogen-Activated Protein Kinase
MDA	malondialdehyde
MIA	Maternal Immune Activation
mitNOS	Mitochondrial Nitric Oxide Synthase (putative)
MPO	myeloperoxidase
mtDNA	mitochondrial DNA
MTHFR	Methylenetetrahydrofolate Reductase
mTOR	Mechanistic Target of Rapamycin
NADPH	Nicotinamide Adenine Dinucleotide Phosphate (reduced form)
NF-κB	Nuclear Factor Kappa-Light-Chain-Enhancer of Activated B Cells
NMDA	N-Methyl-D-Aspartate
NO	Nitric oxide
NOS	Nitric oxide synthase
nNOS/eNOS/iNOS	Neuronal/Endothelial/Inducible Nitric Oxide Synthase
NOX	NOX NADPH oxidase isoform
NRF2	Nuclear factor erythroid 2–related factor 2 (gene)
Nrf2	Nuclear factor erythroid 2–related factor 2 (gene product)
OGG1	8-oxoguanine DNA glycosylase-1
ONOO^−^	Peroxynitrite
PGC-1α	Peroxisome Proliferator-Activated Receptor Gamma Coactivator 1-Alpha
PI3K-Akt	Phosphoinositide 3-Kinase—Protein Kinase B (Akt) (pathway)
poly I:C	Polyinosinic:polycytidylic acid
PON1	Paraoxonase 1
PUFA	Polyunsaturated Fatty Acids
RNS	Reactive Nitrogen Species
ROS	herein Reactive Oxidant Species (not Reactive Oxygen Species)
SAH	S-Adenosylhomocysteine
SAM	S-Adenosylmethionine
SHANK3	SH3 and Multiple Ankyrin Repeat Domains 3 (gene)
Shank3	SH3 and Multiple Ankyrin Repeat Domains 3 (gene product)
SNARE	Soluble N-ethylmaleimide-Sensitive Factor (NSF) Attachment Protein Receptor
SNPs	Single Nucleotide Polymorphisms
SOD	Superoxide Dismutase
TAS2Rs	Taste Receptor Type 2 family (bitter taste receptors)
TLR	Toll-Like Receptors
TNF-α	Tumor Necrosis Factor alpha
UPR	Unfolded protein response
VNTR	Variable Number Tandem Repeat
XO	Xanthine Oxidase

## References

[1] Lord C., Elsabbagh M., Baird G., Veenstra-Vanderweele J. (2018). Autism Spectrum Disorder. Lancet.

[2] American Psychiatric Association (2022). Diagnostic and Statistical Manual of Mental Disorders, Fifth Edition, Text Revision (DSM-5-TR).

[3] Christensen D.L., Baio J., Van Naarden Braun K., Bilder D., Charles J., Constantino J.N., Daniels J., Durkin M.S., Fitzgerald R.T., Kurzius-Spencer M. (2016). Prevalence and Characteristics of Autism Spectrum Disorder Among Children Aged 8 Years-Autism and Developmental Disabilities Monitoring Network, 11 Sites, United States, 2012. MMWR Surveill. Summ..

[4] Loke Y.J., Hannan A.J., Craig J.M. (2015). The Role of Epigenetic Change in Autism Spectrum Disorders. Front. Neurol..

[5] Masini E., Loi E., Vega-Benedetti A.F., Carta M., Doneddu G., Fadda R., Zavattari P. (2020). An Overview of the Main Genetic, Epigenetic and Environmental Factors Involved in Autism Spectrum Disorder Focusing on Synaptic Activity. Int. J. Mol. Sci..

[6] Frye R.E., James S.J. (2014). Metabolic Pathology of Autism in Relation to Redox Metabolism. Biomark. Med..

[7] Kuźniar-Pałka A. (2025). The Role of Oxidative Stress in Autism Spectrum Disorder Pathophysiology, Diagnosis and Treatment. Biomedicines.

[8] Volk H.E., Park B., Hollingue C., Jones K.L., Ashwood P., Windham G.C., Lurman F., Alexeeff S.E., Kharrazi M., Pearl M. (2020). Maternal Immune Response and Air Pollution Exposure during Pregnancy: Insights from the Early Markers for Autism (EMA) Study. J. Neurodev. Disord..

[9] Zoghbi H.Y., Bear M.F. (2012). Synaptic Dysfunction in Neurodevelopmental Disorders Associated with Autism and Intellectual Disabilities. Cold Spring Harb. Perspect. Biol..

[10] Liao X., Liu Y., Fu X., Li Y. (2020). Postmortem Studies of Neuroinflammation in Autism Spectrum Disorder: A Systematic Review. Mol. Neurobiol..

[11] Bjørklund G., Meguid N.A., El-Bana M.A., Tinkov A.A., Saad K., Dadar M., Hemimi M., Skalny A.V., Hosnedlová B., Kizek R. (2020). Oxidative Stress in Autism Spectrum Disorder. Mol. Neurobiol..

[12] Rossignol D.A., Frye R.E. (2014). Evidence Linking Oxidative Stress, Mitochondrial Dysfunction, and Inflammation in the Brain of Individuals with Autism. Front. Physiol..

[13] Bjørklund G., Doşa M.D., Maes M., Dadar M., Frye R.E., Peana M., Chirumbolo S. (2021). The Impact of Glutathione Metabolism in Autism Spectrum Disorder. Pharmacol. Res..

[14] Renaldi R., Persico A.M., Wiguna T., Tanra A.J. (2025). Breaking the Cycle of Oxidative Stress for Better Behavioral Health in Autism Spectrum Disorder: A Scoping Review. Asian J. Psychiatry.

[15] Valko M., Leibfritz D., Moncol J., Cronin M.T.D., Mazur M., Telser J. (2007). Free Radicals and Antioxidants in Normal Physiological Functions and Human Disease. Int. J. Biochem. Cell Biol..

[16] Mormone E., Iorio E.L. (2023). Editorial: Regenerative Medicine in Neurodegenerative Diseases and Aging: Challenging the Redox Homeostasis. Front. Neurosci..

[17] Travagli V., Iorio E.L. (2023). The Biological and Molecular Action of Ozone and Its Derivatives: State-of-the-Art, Enhanced Scenarios, and Quality Insights. Int. J. Mol. Sci..

[18] Macedo Signorelli N.S., Rende S.G.S., Iorio E.L., Ferraz D.C., Paranhos L.R., Moura C.C.G. (2024). Identification of Oxidative Stress Biomarkers in Apical Periodontitis: A Scoping Review with Bibliometric Analysis. Aust. Endod. J..

[19] Chauhan A., Chauhan V. (2006). Oxidative Stress in Autism. Pathophysiology.

[20] Pangrazzi L., Balasco L., Bozzi Y. (2020). Oxidative Stress and Immune System Dysfunction in Autism Spectrum Disorders. Int. J. Mol. Sci..

[21] Lushchak V.I. (2015). Free radicals, reactive oxygen species, oxidative stresses and their classifications. Ukr. Biochem. J..

[22] Forman H.J. (2016). Redox Signaling: An Evolution from Free Radicals to Aging. Free Radic. Biol. Med..

[23] Sies H. (2021). Oxidative Eustress: On Constant Alert for Redox Homeostasis. Redox Biol..

[24] Halliwell B., Gutteridge J.M.C. (2015). Free Radicals in Biology and Medicine.

[25] Pizzino G., Irrera N., Cucinotta M., Pallio G., Mannino F., Arcoraci V., Squadrito F., Altavilla D., Bitto A. (2017). Oxidative Stress: Harms and Benefits for Human Health. Oxidative Med. Cell. Longev..

[26] Bienertova-Vasku J., Lenart P., Scheringer M. (2020). Eustress and Distress: Neither Good Nor Bad, but Rather the Same?. BioEssays.

[27] Su L.-J., Zhang J.-H., Gomez H., Murugan R., Hong X., Xu D., Jiang F., Peng Z.-Y. (2019). Reactive Oxygen Species-Induced Lipid Peroxidation in Apoptosis, Autophagy, and Ferroptosis. Oxidative Med. Cell. Longev..

[28] Nelson D.L., Cox M.M. (2017). Lehninger Principles of Biochemistry.

[29] WEISS J. (1958). One-Electron versus Two-Electron Transfer Processes in the Mechanism of Oxidation-Reduction Reactions in Solution. Nature.

[30] Liochev S.I., Fridovich I. (1994). The Role of O_2_·^−^ in the Production of HO·: In Vitro and in Vivo. Free Radic. Biol. Med..

[31] Rahal A., Kumar A., Singh V., Yadav B., Tiwari R., Chakraborty S., Dhama K. (2014). Oxidative Stress, Prooxidants, and Antioxidants: The Interplay. BioMed Res. Int..

[32] Santilli G., Lamorte G., Carlessi L., Ferrari D., Rota Nodari L., Binda E., Delia D., Vescovi A.L., De Filippis L. (2010). Mild Hypoxia Enhances Proliferation and Multipotency of Human Neural Stem Cells. PLoS ONE.

[33] De Filippis L., Delia D. (2011). Hypoxia in the Regulation of Neural Stem Cells. Cell. Mol. Life Sci..

[34] Mormone E., Iorio E.L., Abate L., Rodolfo C. (2023). Sirtuins and Redox Signaling Interplay in Neurogenesis, Neurodegenerative Diseases, and Neural Cell Reprogramming. Front. Neurosci..

[35] Cobley J.N., Fiorello M.L., Bailey D.M. (2018). 13 Reasons Why the Brain Is Susceptible to Oxidative Stress. Redox Biol..

[36] Wang X., Michaelis E.K. (2010). Selective Neuronal Vulnerability to Oxidative Stress in the Brain. Front. Aging Neurosci..

[37] Murphy M.P., Bayir H., Belousov V., Chang C.J., Davies K.J.A., Davies M.J., Dick T.P., Finkel T., Forman H.J., Janssen-Heininger Y. (2022). Guidelines for Measuring Reactive Oxygen Species and Oxidative Damage in Cells and in Vivo. Nat. Metab..

[38] Ghezzi P., Jaquet V., Marcucci F., Schmidt H.H.H.W. (2017). The Oxidative Stress Theory of Disease: Levels of Evidence and Epistemological Aspects. Br. J. Pharmacol..

[39] Whayne T.F., Saha S.P., Mukherjee D. (2016). Antioxidants in the Practice of Medicine; What Should the Clinician Know?. Cardiovasc. Hematol. Disord. Drug Targets.

[40] Zhuang H., Liang Z., Ma G., Qureshi A., Ran X., Feng C., Liu X., Yan X., Shen L. (2024). Autism Spectrum Disorder: Pathogenesis, Biomarker, and Intervention Therapy. MedComm.

[41] Zhang L., Wang X., Cueto R., Effi C., Zhang Y., Tan H., Qin X., Ji Y., Yang X., Wang H. (2019). Biochemical Basis and Metabolic Interplay of Redox Regulation. Redox Biol..

[42] Buettner G.R. (1993). The Pecking Order of Free Radicals and Antioxidants: Lipid Peroxidation, Alpha-Tocopherol, and Ascorbate. Arch. Biochem. Biophys..

[43] Sies H. (2017). Hydrogen Peroxide as a Central Redox Signaling Molecule in Physiological Oxidative Stress: Oxidative Eustress. Redox Biol..

[44] Ayala A., Muñoz M.F., Argüelles S. (2014). Lipid Peroxidation: Production, Metabolism, and Signaling Mechanisms of Malondialdehyde and 4-Hydroxy-2-Nonenal. Oxidative Med. Cell. Longev..

[45] Gaschler M.M., Stockwell B.R. (2017). Lipid Peroxidation in Cell Death. Biochem. Biophys. Res. Commun..

[46] Jones D.P., Sies H. (2015). The Redox Code. Antioxid. Redox Signal..

[47] Nishida M., Kumagai Y., Ihara H., Fujii S., Motohashi H., Akaike T. (2016). Redox Signaling Regulated by Electrophiles and Reactive Sulfur Species. J. Clin. Biochem. Nutr..

[48] Davinelli S., Medoro A., Siracusano M., Savino R., Saso L., Scapagnini G., Mazzone L. (2025). Oxidative Stress Response and NRF2 Signaling Pathway in Autism Spectrum Disorder. Redox Biol..

[49] Cadenas E., Packer L., Traber M.G. (2016). Antioxidants, Oxidants, and Redox Impacts on Cell Function—A Tribute to Helmut Sies. Arch. Biochem. Biophys..

[50] Sevanian A., Muakkassah-Kelly S.F., Montestruque S. (1983). The Influence of Phospholipase A2 and Glutathione Peroxidase on the Elimination of Membrane Lipid Peroxides. Arch. Biochem. Biophys..

[51] Liu X., Lin J., Zhang H., Khan N.U., Zhang J., Tang X., Cao X., Shen L. (2022). Oxidative Stress in Autism Spectrum Disorder-Current Progress of Mechanisms and Biomarkers. Front. Psychiatry.

[52] Bedard K., Krause K.-H. (2007). The NOX Family of ROS-Generating NADPH Oxidases: Physiology and Pathophysiology. Physiol. Rev..

[53] Fang J., Sheng R., Qin Z.-H. (2021). NADPH Oxidases in the Central Nervous System: Regional and Cellular Localization and the Possible Link to Brain Diseases. Antioxid. Redox Signal..

[54] Zhang X., Ibi M., Haga R., Iwata K., Matsumoto M., Asaoka N., Liu J., Katsuyama M., Yabe-Nishimura C. (2021). NOX1/NADPH Oxidase Affects the Development of Autism-like Behaviors in a Maternal Immune Activation Model. Biochem. Biophys. Res. Commun..

[55] Solanki K., Rajpoot S., Bezsonov E.E., Orekhov A.N., Saluja R., Wary A., Axen C., Wary K., Baig M.S. (2022). The Expanding Roles of Neuronal Nitric Oxide Synthase (NOS1). PeerJ.

[56] Jones D.N., Raghanti M.A. (2021). The Role of Monoamine Oxidase Enzymes in the Pathophysiology of Neurological Disorders. J. Chem. Neuroanat..

[57] Gu F., Chauhan V., Chauhan A. (2017). Monoamine Oxidase-A and B Activities in the Cerebellum and Frontal Cortex of Children and Young Adults with Autism. J. Neurosci. Res..

[58] Yui K., Imataka G., Yoshihara S. (2022). Lipid-Based Molecules on Signaling Pathways in Autism Spectrum Disorder. Int. J. Mol. Sci..

[59] El-Ansary A., Al-Ayadhi L. (2012). Lipid Mediators in Plasma of Autism Spectrum Disorders. Lipids Health Dis..

[60] Liu Y., Sun Y., Chen A., Chen J., Zhu T., Wang S., Qiao W., Zhou D., Zhang X., Chen S. (2025). Involvement of Disulfidptosis in the Pathophysiology of Autism Spectrum Disorder. Life Sci..

[61] Zheng Y., Sun J., Luo Z., Li Y., Huang Y. (2024). Emerging Mechanisms of Lipid Peroxidation in Regulated Cell Death and Its Physiological Implications. Cell Death Dis..

[62] Ayoub G. (2024). Neurodevelopment of Autism: Critical Periods, Stress and Nutrition. Cells.

[63] Buss C. (2021). Maternal Oxidative Stress during Pregnancy and Offspring Neurodevelopment. Brain Behav. Immun..

[64] Carpita B., Muti D., Dell’Osso L. (2018). Oxidative Stress, Maternal Diabetes, and Autism Spectrum Disorders. Oxidative Med. Cell. Longev..

[65] Marzola P., Melzer T., Pavesi E., Gil-Mohapel J., Brocardo P.S. (2023). Exploring the Role of Neuroplasticity in Development, Aging, and Neurodegeneration. Brain Sci..

[66] Modabbernia A., Velthorst E., Reichenberg A. (2017). Environmental Risk Factors for Autism: An Evidence-Based Review of Systematic Reviews and Meta-Analyses. Mol. Autism.

[67] Panisi C., Guerini F.R., Abruzzo P.M., Balzola F., Biava P.M., Bolotta A., Brunero M., Burgio E., Chiara A., Clerici M. (2021). Autism Spectrum Disorder from the Womb to Adulthood: Suggestions for a Paradigm Shift. J. Pers. Med..

[68] Singh S., Goel I., Tripathi S., Ahirwar A., Kumar M., Rana A., Dhar R., Karmakar S. (2024). Effect of Environmental Air Pollutants on Placental Function and Pregnancy Outcomes: A Molecular Insight. Environ. Sci. Pollut. Res. Int..

[69] Toscano C.V.A., Barros L., Lima A.B., Nunes T., Carvalho H.M., Gaspar J.M. (2021). Neuroinflammation in Autism Spectrum Disorders: Exercise as a “Pharmacological” Tool. Neurosci. Biobehav. Rev..

[70] She K., Yuan N., Huang M., Zhu W., Tang M., Ma Q., Chen J. (2024). Emerging Role of Microglia in the Developing Dopaminergic System: Perturbation by Early Life Stress. Neural Regen. Res..

[71] Meguid N.A., Dardir A.A., Abdel-Raouf E.R., Hashish A. (2011). Evaluation of Oxidative Stress in Autism: Defective Antioxidant Enzymes and Increased Lipid Peroxidation. Biol. Trace Elem. Res..

[72] Frye R.E., Rose S., Voinsky I., Gurwitz D. (2024). Nitrosative Stress in Autism: Supportive Evidence and Implications for Mitochondrial Dysfunction. Adv. Sci..

[73] Burke S.L., Cobb J., Agarwal R., Maddux M., Cooke M.S. (2021). How Robust Is the Evidence for a Role of Oxidative Stress in Autism Spectrum Disorders and Intellectual Disabilities?. J. Autism Dev. Disord..

[74] Li B., Ming H., Qin S., Nice E.C., Dong J., Du Z., Huang C. (2025). Redox Regulation: Mechanisms, Biology and Therapeutic Targets in Diseases. Signal Transduct. Target. Ther..

[75] Pal A., Goel F., Sharma A., Garg V.K. (2025). Oxidative Stress and Antioxidant Therapeutics in Autism Spectrum Disorder: A Biochemical and Structure-Activity Relationship Perspective. Mol. Divers..

[76] Yui K., Kawasaki Y., Yamada H., Ogawa S. (2016). Oxidative Stress and Nitric Oxide in Autism Spectrum Disorder and Other Neuropsychiatric Disorders. CNS Neurol. Disord. Drug Targets.

[77] Delattre J. (2006). [Introduction: From molecular oxygen to oxidative stress and radical biochemistry]. Ann. Pharm. Fr..

[78] Mink J.W., Blumenschine R.J., Adams D.B. (1981). Ratio of Central Nervous System to Body Metabolism in Vertebrates: Its Constancy and Functional Basis. Am. J. Physiol..

[79] Magistretti P.J., Allaman I. (2015). A Cellular Perspective on Brain Energy Metabolism and Functional Imaging. Neuron.

[80] Fornstedt Wallin B. (2024). Oxidation of Dopamine and Related Catechols in Dopaminergic Brain Regions in Parkinson’s Disease and during Ageing in Non-Parkinsonian Subjects. J. Neural Transm..

[81] Umek N., Geršak B., Vintar N., Šoštarič M., Mavri J. (2018). Dopamine Autoxidation Is Controlled by Acidic PH. Front. Mol. Neurosci..

[82] Badillo-Ramírez I., Saniger J.M., Rivas-Arancibia S. (2019). 5-S-Cysteinyl-Dopamine, a Neurotoxic Endogenous Metabolite of Dopamine: Implications for Parkinson’s Disease. Neurochem. Int..

[83] Marotta R., Risoleo M.C., Messina G., Parisi L., Carotenuto M., Vetri L., Roccella M. (2020). The Neurochemistry of Autism. Brain Sci..

[84] Pavăl D. (2023). The Dopamine Hypothesis of Autism Spectrum Disorder: A Comprehensive Analysis of the Evidence. Int. Rev. Neurobiol..

[85] Bortolato M., Godar S.C., Alzghoul L., Zhang J., Darling R.D., Simpson K.L., Bini V., Chen K., Wellman C.L., Lin R.C.S. (2013). Monoamine Oxidase A and A/B Knockout Mice Display Autistic-like Features. Int. J. Neuropsychopharmacol..

[86] Koppenol W.H. (1993). The Centennial of the Fenton Reaction. Free Radic. Biol. Med..

[87] Carocci A., Catalano A., Sinicropi M.S., Genchi G. (2018). Oxidative Stress and Neurodegeneration: The Involvement of Iron. Biometals.

[88] Santos G., Borges J.M.P., Avila-Rodriguez M., Gaíno S.B., Barreto G.E., Rúbio É.P., Aguiar R.M., Galembeck E., Bromochenkel C.B., de Oliveira D.M. (2019). Copper and Neurotoxicity in Autism Spectrum Disorder. Curr. Pharm. Des..

[89] Wang Y., Li D., Xu K., Wang G., Zhang F. (2025). Copper Homeostasis and Neurodegenerative Diseases. Neural Regen. Res..

[90] Zecca L., Stroppolo A., Gatti A., Tampellini D., Toscani M., Gallorini M., Giaveri G., Arosio P., Santambrogio P., Fariello R.G. (2004). The Role of Iron and Copper Molecules in the Neuronal Vulnerability of Locus Coeruleus and Substantia Nigra during Aging. Proc. Natl. Acad. Sci. USA.

[91] Gangania M.K., Batra J., Kushwaha S., Agarwal R. (2017). Role of Iron and Copper in the Pathogenesis of Parkinson’s Disease. Indian J. Clin. Biochem..

[92] Deibel M.A., Ehmann W.D., Markesbery W.R. (1996). Copper, Iron, and Zinc Imbalances in Severely Degenerated Brain Regions in Alzheimer’s Disease: Possible Relation to Oxidative Stress. J. Neurol. Sci..

[93] Zhou Y., Gao J. (2022). Why Not Try to Predict Autism Spectrum Disorder with Crucial Biomarkers in Cuproptosis Signaling Pathway?. Front. Psychiatry.

[94] Oshodi Y., Ojewunmi O., Oshodi T.A., Ijarogbe G.T., Ogun O.C., Aina O.F., Lesi F. (2017). Oxidative Stress Markers and Genetic Polymorphisms of Glutathione S-Transferase T1, M1, and P1 in a Subset of Children with Autism Spectrum Disorder in Lagos, Nigeria. Niger. J. Clin. Pract..

[95] Katsuyama M., Matsuno K., Yabe-Nishimura C. (2012). Physiological Roles of NOX/NADPH Oxidase, the Superoxide-Generating Enzyme. J. Clin. Biochem. Nutr..

[96] Nisimoto Y., Jackson H.M., Ogawa H., Kawahara T., Lambeth J.D. (2010). Constitutive NADPH-Dependent Electron Transferase Activity of the Nox4 Dehydrogenase Domain. Biochemistry.

[97] Braughler J.M., Duncan L.A., Chase R.L. (1986). The Involvement of Iron in Lipid Peroxidation. Importance of Ferric to Ferrous Ratios in Initiation. J. Biol. Chem..

[98] Yui K., Imataka G., Shiohama T. (2023). Lipid NOSation of the Docosahexaenoic Acid/Arachidonic Acid Ratio Relating to the Social Behaviors of Individuals with Autism Spectrum Disorder: The Relationship with Ferroptosis. Int. J. Mol. Sci..

[99] Rose S., Melnyk S., Pavliv O., Bai S., Nick T.G., Frye R.E., James S.J. (2012). Evidence of Oxidative Damage and Inflammation Associated with Low Glutathione Redox Status in the Autism Brain. Transl. Psychiatry.

[100] Frustaci A., Neri M., Cesario A., Adams J.B., Domenici E., Dalla Bernardina B., Bonassi S. (2012). Oxidative Stress-Related Biomarkers in Autism: Systematic Review and Meta-Analyses. Free Radic. Biol. Med..

[101] Liu L., Lai Y., Zhan Z., Fu Q., Jiang Y. (2022). Identification of Ferroptosis-Related Molecular Clusters and Immune Characterization in Autism Spectrum Disorder. Front. Genet..

[102] Rezzani R., Gianò M., Pinto D., Rinaldi F., van Noorden C.J.F., Favero G. (2024). Hepatic Alterations in a BTBR T + Itpr3tf/J Mouse Model of Autism and Improvement Using Melatonin via Mitigation Oxidative Stress, Inflammation and Ferroptosis. Int. J. Mol. Sci..

[103] Wu H., Luan Y., Wang H., Zhang P., Liu S., Wang P., Cao Y., Sun H., Wu L. (2022). Selenium Inhibits Ferroptosis and Ameliorates Autistic-like Behaviors of BTBR Mice by Regulating the Nrf2/GPx4 Pathway. Brain Res. Bull..

[104] Davies M.J., Hawkins C.L. (2020). The Role of Myeloperoxidase in Biomolecule Modification, Chronic Inflammation, and Disease. Antioxid. Redox Signal..

[105] Ray R.S., Katyal A. (2016). Myeloperoxidase: Bridging the Gap in Neurodegeneration. Neurosci. Biobehav. Rev..

[106] Gao C., Jiang J., Tan Y., Chen S. (2023). Microglia in Neurodegenerative Diseases: Mechanism and Potential Therapeutic Targets. Signal Transduct. Target. Ther..

[107] Chen S., Pan J., Gong Z., Wu M., Zhang X., Chen H., Yang D., Qi S., Peng Y., Shen J. (2024). Hypochlorous Acid Derived from Microglial Myeloperoxidase Could Mediate High-Mobility Group Box 1 Release from Neurons to Amplify Brain Damage in Cerebral Ischemia-Reperfusion Injury. J. Neuroinflamm..

[108] Ceylan M.F., Tural Hesapcioglu S., Yavas C.P., Senat A., Erel O. (2021). Serum Ischemia-Modified Albumin Levels, Myeloperoxidase Activity and Peripheral Blood Mononuclear Cells in Autism Spectrum Disorder (ASD). J. Autism Dev. Disord..

[109] Haslacher H., Perkmann T., Lukas I., Barth A., Ponocny-Seliger E., Michlmayr M., Scheichenberger V., Wagner O., Winker R. (2012). Myeloperoxidase Levels Predict Executive Function. Int. J. Sports Med..

[110] Nguyen K.D., Qiu Y., Cui X., Goh Y.P.S., Mwangi J., David T., Mukundan L., Brombacher F., Locksley R.M., Chawla A. (2011). Alternatively Activated Macrophages Produce Catecholamines to Sustain Adaptive Thermogenesis. Nature.

[111] Mor G., Yue W., Santen R.J., Gutierrez L., Eliza M., Berstein L.M., Harada N., Wang J., Lysiak J., Diano S. (1998). Macrophages, Estrogen and the Microenvironment of Breast Cancer. J. Steroid Biochem. Mol. Biol..

[112] Alderton W.K., Cooper C.E., Knowles R.G. (2001). Nitric Oxide Synthases: Structure, Function and Inhibition. Biochem. J..

[113] Filho C.C., Melfior L., Ramos S.L., Pizi M.S.O., Taruhn L.F., Muller M.E., Nunes T.K., Schmitt L.d.O., Gaspar J.M., de Oliveira M.d.A. (2025). Tetrahydrobiopterin and Autism Spectrum Disorder: A Systematic Review of a Promising Therapeutic Pathway. Brain Sci..

[114] Haynes V., Elfering S., Traaseth N., Giulivi C. (2004). Mitochondrial Nitric-Oxide Synthase: Enzyme Expression, Characterization, and Regulation. J. Bioenerg. Biomembr..

[115] Brown G.C. (1999). Nitric Oxide and Mitochondrial Respiration. Biochim. Biophys. Acta.

[116] Marks J.D., Boriboun C., Wang J. (2005). Mitochondrial Nitric Oxide Mediates Decreased Vulnerability of Hippocampal Neurons from Immature Animals to NMDA. J. Neurosci..

[117] Okamoto S., Lipton S.A. (2015). S-Nitrosylation in Neurogenesis and Neuronal Development. Biochim. Biophys. Acta.

[118] Kapil V., Khambata R.S., Jones D.A., Rathod K., Primus C., Massimo G., Fukuto J.M., Ahluwalia A. (2020). The Noncanonical Pathway for In Vivo Nitric Oxide Generation: The Nitrate-Nitrite-Nitric Oxide Pathway. Pharmacol. Rev..

[119] Bortolotti M., Polito L., Battelli M.G., Bolognesi A. (2021). Xanthine Oxidoreductase: One Enzyme for Multiple Physiological Tasks. Redox Biol..

[120] Tripathi M.K., Kartawy M., Amal H. (2020). The Role of n itric Oxide in Brain Disorders: Autism Spectrum Disorder and Other Psychiatric, Neurological, and Neurodegenerative Disorders. Redox Biol..

[121] Khan A.A., Dewald H.D. (2025). Nitric Oxide and Peroxynitrite as New Biomarkers for Early Diagnosis of Autism. Brain Res..

[122] Sajdel-Sulkowska E.M., Lipinski B., Windom H., Audhya T., McGinnis W. (2008). Oxidative Stress in Autism: Elevated Cerebellar 3-Nitrotyrosine Levels. Am. J. Biochem. Biotechnol..

[123] Al-Garni A.M., Hosny S.A., Almasabi F., Shati A.A., Alzamil N.M., ShamsEldeen A.M., El-Shafei A.A., Al-Hashem F., Zafrah H., Maarouf A. (2025). Identifying INOS and Glycogen as Biomarkers for Degenerated Cerebellar Purkinje Cells in Autism Spectrum Disorder: Protective Effects of Erythropoietin and Zinc Sulfate. PLoS ONE.

[124] Tripathi M.K., Ojha S.K., Kartawy M., Hamoudi W., Choudhary A., Stern S., Aran A., Amal H. (2023). The NO Answer for Autism Spectrum Disorder. Adv. Sci..

[125] Steinert J.R., Amal H. (2023). The Contribution of an Imbalanced Redox Signalling to Neurological and Neurodegenerative Conditions. Free Radic. Biol. Med..

[126] Khudhur Z.O., Abdullah S.R., Hussen B.M., Murad N.A., Sayad A., Ghafouri-Fard S. (2025). Gasotransmitters and Their Influence on Autism Spectrum Disorders—A Systematic Review. Mol. Biol. Rep..

[127] Whitfield J.B. (2001). Gamma Glutamyl Transferase. Crit. Rev. Clin. Lab. Sci..

[128] Hanigan M.H., Frierson H.F.J. (1996). Immunohistochemical Detection of Gamma-Glutamyl Transpeptidase in Normal Human Tissue. J. Histochem. Cytochem..

[129] Tzeng Y.-Z., Hu C.-H. (2014). Radical-Induced Cis-Trans Isomerization of Fatty Acids: A Theoretical Study. J. Phys. Chem. A.

[130] Chatgilialoglu C., Zambonin L., Altieri A., Ferreri C., Mulazzani Q.G., Landi L. (2002). Geometrical Isomerism of Monounsaturated Fatty Acids: Thiyl Radical Catalysis and Influence of Antioxidant Vitamins. Free Radic. Biol. Med..

[131] Accaoui M.J., Enoiu M., Mergny M., Masson C., Dominici S., Wellman M., Visvikis A. (2000). Gamma-Glutamyltranspeptidase-Dependent Glutathione Catabolism Results in Activation of NF-KB. Biochem. Biophys. Res. Commun..

[132] Paolicchi A., Minotti G., Tonarelli P., Tongiani R., De Cesare D., Mezzetti A., Dominici S., Comporti M., Pompella A. (1999). Gamma-Glutamyl Transpeptidase-Dependent Iron Reduction and LDL Oxidation—A Potential Mechanism in Atherosclerosis. J. Investig. Med..

[133] Li S., Liao X., Pan Y., Xiang X., Zhang Y. (2022). Gamma-Glutamyl Transferase Levels Are Associated with the Occurrence of Post-Stroke Cognitive Impairment: A Multicenter Cohort Study. BMC Neurol..

[134] Starkov A.A., Fiskum G., Chinopoulos C., Lorenzo B.J., Browne S.E., Patel M.S., Beal M.F. (2004). Mitochondrial Alpha-Ketoglutarate Dehydrogenase Complex Generates Reactive Oxygen Species. J. Neurosci..

[135] Jodeiri Farshbaf M., Kiani-Esfahani A. (2018). Succinate Dehydrogenase: Prospect for Neurodegenerative Diseases. Mitochondrion.

[136] Mrácek T., Pecinová A., Vrbacký M., Drahota Z., Houstek J. (2009). High Efficiency of ROS Production by Glycerophosphate Dehydrogenase in Mammalian Mitochondria. Arch. Biochem. Biophys..

[137] Vasquez-Vivar J., Kalyanaraman B., Kennedy M.C. (2000). Mitochondrial Aconitase Is a Source of Hydroxyl Radical. An Electron Spin Resonance Investigation. J. Biol. Chem..

[138] Hey-Mogensen M., Goncalves R.L.S., Orr A.L., Brand M.D. (2014). Production of Superoxide/H_2_O_2_ by Dihydroorotate Dehydrogenase in Rat Skeletal Muscle Mitochondria. Free Radic. Biol. Med..

[139] Fox R.J., Wiendl H., Wolf C., De Stefano N., Sellner J., Gryb V., Rejdak K., Bozhinov P.S., Tomakh N., Skrypchenko I. (2022). A Double-Blind, Randomized, Placebo-Controlled Phase 2 Trial Evaluating the Selective Dihydroorotate Dehydrogenase Inhibitor Vidofludimus Calcium in Relapsing-Remitting Multiple Sclerosis. Ann. Clin. Transl. Neurol..

[140] Sabol S.Z., Hu S., Hamer D. (1998). A Functional Polymorphism in the Monoamine Oxidase A Gene Promoter. Hum. Genet..

[141] Harneit A., Braun U., Geiger L.S., Zang Z., Hakobjan M., van Donkelaar M.M.J., Schweiger J.I., Schwarz K., Gan G., Erk S. (2019). MAOA-VNTR Genotype Affects Structural and Functional Connectivity in Distributed Brain Networks. Hum. Brain Mapp..

[142] Shih J.C., Chen K., Ridd M.J. (1999). Monoamine Oxidase: From Genes to Behavior. Annu. Rev. Neurosci..

[143] Wang C.C., Billett E., Borchert A., Kuhn H., Ufer C. (2013). Monoamine Oxidases in Development. Cell. Mol. Life Sci..

[144] Wanders R.J.A., Baes M., Ribeiro D., Ferdinandusse S., Waterham H.R. (2023). The Physiological Functions of Human Peroxisomes. Physiol. Rev..

[145] Wanders R.J.A., Waterham H.R. (2005). Peroxisomal Disorders I: Biochemistry and Genetics of Peroxisome Biogenesis Disorders. Clin. Genet..

[146] Sarkar C., Lipinski M.M. (2024). Role and Function of Peroxisomes in Neuroinflammation. Cells.

[147] El-Ansary A., Alhakbany M., Aldbass A., Qasem H., Al-Mazidi S., Bhat R.S., Al-Ayadhi L. (2021). Alpha-Synuclein, Cyclooxygenase-2 and Prostaglandins-EP2 Receptors as Neuroinflammatory Biomarkers of Autism Spectrum Disorders: Use of Combined ROC Curves to Increase Their Diagnostic Values. Lipids Health Dis..

[148] Zhao G., Gao J., Liang S., Wang X., Sun C., Xia W., Hao Y., Li X., Cao Y., Wu L. (2015). Study of the Serum Levels of Polyunsaturated Fatty Acids and the Expression of Related Liver Metabolic Enzymes in a Rat Valproate-Induced Autism Model. Int. J. Dev. Neurosci..

[149] Sun C., Zou M., Wang X., Xia W., Ma Y., Liang S., Hao Y., Wu L., Fu S. (2018). FADS1-FADS2 and ELOVL2 Gene Polymorphisms in Susceptibility to Autism Spectrum Disorders in Chinese Children. BMC Psychiatry.

[150] Yui K., Imataka G., Ichihashi M. (2025). Prostaglandins: Biological Action, Therapeutic Aspects, and Pathophysiology of Autism Spectrum Disorders. Curr. Issues Mol. Biol..

[151] Gasser B.A., Kurz J., Dick B., Mohaupt M.G. (2022). How Is CYP17A1 Activity Altered in Autism? A Pilot Study to Identify Potential Pharmacological Targets. Life.

[152] Durairaj P., Liu Z.L. (2025). Brain Cytochrome P_450_: Navigating Neurological Health and Metabolic Regulation. J. Xenobiot..

[153] Ballester P., Espadas C., Almenara S., Barrachina J., Muriel J., Ramos E., Toral N., Belda C., Peiró A.M. (2023). CYP2D6 Genotype and Pharmacovigilance Impact on Autism Spectrum Disorder: A Naturalistic Study with Extreme Phenotype Analysis. Pharmaceuticals.

[154] Dhulkifle H., Agouni A., Zeidan A., Al-Kuwari M.S., Parray A., Tolefat M., Korashy H.M. (2021). Influence of the Aryl Hydrocarbon Receptor Activating Environmental Pollutants on Autism Spectrum Disorder. Int. J. Mol. Sci..

[155] Harrison R. (2002). Structure and Function of Xanthine Oxidoreductase: Where Are We Now?. Free Radic. Biol. Med..

[156] Godber B.L., Doel J.J., Sapkota G.P., Blake D.R., Stevens C.R., Eisenthal R., Harrison R. (2000). Reduction of Nitrite to Nitric Oxide Catalyzed by Xanthine Oxidoreductase. J. Biol. Chem..

[157] Thorsen M., Bilenberg N., Thorsen L., Michel T.M. (2022). Oxidative Stress in Adults with Autism Spectrum Disorder: A Case Control Study. J. Autism Dev. Disord..

[158] Zoroglu S.S., Armutcu F., Ozen S., Gurel A., Sivasli E., Yetkin O., Meram I. (2004). Increased Oxidative Stress and Altered Activities of Erythrocyte Free Radical Scavenging Enzymes in Autism. Eur. Arch. Psychiatry Clin. Neurosci..

[159] Dong D., Zielke H.R., Yeh D., Yang P. (2018). Cellular Stress and Apoptosis Contribute to the Pathogenesis of Autism Spectrum Disorder. Autism Res..

[160] Badawy A.A.-B. (2020). Kynurenine Pathway and Human Systems. Exp. Gerontol..

[161] Bryn V., Verkerk R., Skjeldal O.H., Saugstad O.D., Ormstad H. (2017). Kynurenine Pathway in Autism Spectrum Disorders in Children. Neuropsychobiology.

[162] Notarangelo F.M., Pocivavsek A. (2017). Elevated Kynurenine Pathway Metabolism during Neurodevelopment: Implications for Brain and Behavior. Neuropharmacology.

[163] Santana-Coelho D. (2024). Does the Kynurenine Pathway Play a Pathogenic Role in Autism Spectrum Disorder?. Brain Behav. Immun.-Health.

[164] Le Belle J.E., Sperry J., Ngo A., Ghochani Y., Laks D.R., López-Aranda M., Silva A.J., Kornblum H.I. (2014). Maternal Inflammation Contributes to Brain Overgrowth and Autism-Associated Behaviors through Altered Redox Signaling in Stem and Progenitor Cells. Stem Cell Rep..

[165] Nadeem A., Ahmad S.F., Al-Harbi N.O., Attia S.M., Alshammari M.A., Alzahrani K.S., Bakheet S.A. (2019). Increased Oxidative Stress in the Cerebellum and Peripheral Immune Cells Leads to Exaggerated Autism-like Repetitive Behavior Due to Deficiency of Antioxidant Response in BTBR T + tf/J Mice. Prog. Neuro-Psychopharmacol. Biol. Psychiatry.

[166] Walton J.C., Selvakumar B., Weil Z.M., Snyder S.H., Nelson R.J. (2013). Neuronal Nitric Oxide Synthase and NADPH Oxidase Interact to Affect Cognitive, Affective, and Social Behaviors in Mice. Behav. Brain Res..

[167] Ming X., Stein T.P., Brimacombe M., Johnson W.G., Lambert G.H., Wagner G.C. (2005). Increased Excretion of a Lipid Peroxidation Biomarker in Autism. Prostaglandins Leukot. Essent. Fat. Acids.

[168] Uchida K. (2003). 4-Hydroxy-2-Nonenal: A Product and Mediator of Oxidative Stress. Prog. Lipid Res..

[169] Wu X., Li R., Hong Q., Chi X. (2022). Development and Validation of a Novel Diagnostic Model for Childhood Autism Spectrum Disorder Based on Ferroptosis-Related Genes. Front. Psychiatry.

[170] Bjørklund G., Tinkov A.A., Hosnedlová B., Kizek R., Ajsuvakova O.P., Chirumbolo S., Skalnaya M.G., Peana M., Dadar M., El-Ansary A. (2020). The Role of Glutathione Redox Imbalance in Autism Spectrum Disorder: A Review. Free Radic. Biol. Med..

[171] Wu H., Zhao G., Liu S., Zhang Q., Wang P., Cao Y., Wu L. (2022). Supplementation with Selenium Attenuates Autism-like Behaviors and Improves Oxidative Stress, Inflammation and Related Gene Expression in an Autism Disease Model. J. Nutr. Biochem..

[172] Yui K., Imataka G., Shiohama T. (2023). Lipid Peroxidation via Regulating the Metabolism of Docosahexaenoic Acid and Arachidonic Acid in Autistic Behavioral Symptoms. Curr. Issues Mol. Biol..

[173] Zambonin L., Ferreri C., Cabrini L., Prata C., Chatgilialoglu C., Landi L. (2006). Occurrence of Trans Fatty Acids in Rats Fed a Trans-Free Diet: A Free Radical-Mediated Formation?. Free Radic. Biol. Med..

[174] Neish A.S., Jones R.M. (2014). Redox Signaling Mediates Symbiosis between the Gut Microbiota and the Intestine. Gut Microbes.

[175] Trostchansky A., Mastrogiovanni M., Miquel E., Rodríguez-Bottero S., Martínez-Palma L., Cassina P., Rubbo H. (2018). Profile of Arachidonic Acid-Derived Inflammatory Markers and Its Modulation by Nitro-Oleic Acid in an Inherited Model of Amyotrophic Lateral Sclerosis. Front. Mol. Neurosci..

[176] Hung W.-L., Sun Hwang L., Shahidi F., Pan M.-H., Wang Y., Ho C.-T. (2016). Endogenous Formation of Trans Fatty Acids: Health Implications and Potential Dietary Intervention. J. Funct. Foods.

[177] de Souza R.J., Mente A., Maroleanu A., Cozma A.I., Ha V., Kishibe T., Uleryk E., Budylowski P., Schünemann H., Beyene J. (2015). Intake of Saturated and Trans Unsaturated Fatty Acids and Risk of All Cause Mortality, Cardiovascular Disease, and Type 2 Diabetes: Systematic Review and Meta-Analysis of Observational Studies. BMJ.

[178] Schilter D. (2017). Thiol Oxidation: A Slippery Slope. Nat. Rev. Chem..

[179] Winterbourn C.C. (2020). Hydrogen Peroxide Reactivity and Specificity in Thiol-Based Cell Signalling. Biochem. Soc. Trans..

[180] Schrier M.S., Zhang Y., Trivedi M.S., Deth R.C. (2022). Decreased Cortical Nrf2 Gene Expression in Autism and Its Relationship to Thiol and Cobalamin Status. Biochimie.

[181] Frye R.E., Rincon N., McCarty P.J., Brister D., Scheck A.C., Rossignol D.A. (2024). Biomarkers of Mitochondrial Dysfunction in Autism Spectrum Disorder: A Systematic Review and Meta-Analysis. Neurobiol. Dis..

[182] Kim S., Kim H., Yim Y.S., Ha S., Atarashi K., Tan T.G., Longman R.S., Honda K., Littman D.R., Choi G.B. (2017). Maternal Gut Bacteria Promote Neurodevelopmental Abnormalities in Mouse Offspring. Nature.

[183] Napoli E., Wong S., Hertz-Picciotto I., Giulivi C. (2014). Deficits in Bioenergetics and Impaired Immune Response in Granulocytes from Children with Autism. Pediatrics.

[184] Ayaydın H., Kılıçaslan F., Koyuncu İ., Çelik H., Çalık M., Güzelçiçek A., Kirmit A. (2021). Impaired Thiol/Disulfide Homeostasis in Children Diagnosed with Autism: A Case-Control Study. J. Mol. Neurosci..

[185] Teke H., Balci S., Neselioglu S., Teke S., Erel O., Tamer L., Toros F. (2025). Oxidative Stress and Dynamic Thiol/Disulfide Homeostasis in Autism: A Focus on Early Childhood. J. Mol. Neurosci..

[186] Efe A., Neşelioğlu S., Soykan A. (2021). An Investigation of the Dynamic Thiol/Disulfide Homeostasis, As a Novel Oxidative Stress Plasma Biomarker, in Children With Autism Spectrum Disorders. Autism Res..

[187] Cortés E., Aguilar M.J., Rizo M.M., Gil V., Hidalgo M. (2013). Trans Fatty Acids in the Nutrition of Children with Neurological Disorders. Nutr. Hosp..

[188] Hamoudi W., Tripathi M.K., Ojha S.K., Amal H. (2022). A Cross-Talk between Nitric Oxide and the Glutamatergic System in a Shank3 Mouse Model of Autism. Free Radic. Biol. Med..

[189] Kang K.W., Choi S.H., Kim S.G. (2002). Peroxynitrite Activates NF-E2-Related Factor 2/Antioxidant Response Element through the Pathway of Phosphatidylinositol 3-Kinase: The Role of Nitric Oxide Synthase in Rat Glutathione S-Transferase A2 Induction. Nitric Oxide Biol. Chem..

[190] Nadeem A., Ahmad S.F., Al-Ayadhi L.Y., Attia S.M., Al-Harbi N.O., Alzahrani K.S., Bakheet S.A. (2020). Differential Regulation of Nrf2 Is Linked to Elevated Inflammation and Nitrative Stress in Monocytes of Children with Autism. Psychoneuroendocrinology.

[191] Söğüt S., Zoroğlu S.S., Ozyurt H., Yilmaz H.R., Ozuğurlu F., Sivasli E., Yetkin O., Yanik M., Tutkun H., Savaş H.A. (2003). Changes in Nitric Oxide Levels and Antioxidant Enzyme Activities May Have a Role in the Pathophysiological Mechanisms Involved in Autism. Clin. Chim. Acta.

[192] Feng C., Chen Y., Pan J., Yang A., Niu L., Min J., Meng X., Liao L., Zhang K., Shen L. (2017). Redox Proteomic Identification of Carbonylated Proteins in Autism Plasma: Insight into Oxidative Stress and Its Related Biomarkers in Autism. Clin. Proteom..

[193] Hu T., Dong Y., He C., Zhao M., He Q. (2020). The Gut Microbiota and Oxidative Stress in Autism Spectrum Disorders (ASD). Oxidative Med. Cell. Longev..

[194] Rose S., Frye R.E., Slattery J., Wynne R., Tippett M., Pavliv O., Melnyk S., James S.J. (2014). Oxidative Stress Induces Mitochondrial Dysfunction in a Subset of Autism Lymphoblastoid Cell Lines in a Well-Matched Case Control Cohort. PLoS ONE.

[195] Rose S., Bennuri S.C., Wynne R., Melnyk S., James S.J., Frye R.E. (2017). Mitochondrial and Redox Abnormalities in Autism Lymphoblastoid Cells: A Sibling Control Study. FASEB J..

[196] Osredkar J., Kumer K., Fabjan T., Jekovec Vrhovšek M., Maček J., Zupan M., Bobrowska-Korczak B., Gątarek P., Rosiak A., Giebułtowicz J. (2023). Determination of Modified Nucleosides in the Urine of Children with Autism Spectrum Disorder. Acta Biochim. Pol..

[197] Melnyk S., Fuchs G.J., Schulz E., Lopez M., Kahler S.G., Fussell J.J., Bellando J., Pavliv O., Rose S., Seidel L. (2012). Metabolic Imbalance Associated with Methylation Dysregulation and Oxidative Damage in Children with Autism. J. Autism Dev. Disord..

[198] Zheng T., Liu Q., Xing F., Zeng C., Wang W. (2023). Disulfidptosis: A New Form of Programmed Cell Death. J. Exp. Clin. Cancer Res..

[199] Howsmon D.P., Kruger U., Melnyk S., James S.J., Hahn J. (2017). Classification and Adaptive Behavior Prediction of Children with Autism Spectrum Disorder Based upon Multivariate Data Analysis of Markers of Oxidative Stress and DNA Methylation. PLoS Comput. Biol..

[200] Li Y., Qiu S., Shi J., Guo Y., Li Z., Cheng Y., Liu Y. (2020). Association between MTHFR C677T/A1298C and Susceptibility to Autism Spectrum Disorders: A Meta-Analysis. BMC Pediatr..

[201] Pu D., Shen Y., Wu J. (2013). Association between MTHFR Gene Polymorphisms and the Risk of Autism Spectrum Disorders: A Meta-Analysis. Autism Res..

[202] Schmidt R.J., Tancredi D.J., Ozonoff S., Hansen R.L., Hartiala J., Allayee H., Schmidt L.C., Tassone F., Hertz-Picciotto I. (2012). Maternal Periconceptional Folic Acid Intake and Risk of Autism Spectrum Disorders and Developmental Delay in the CHARGE (CHildhood Autism Risks from Genetics and Environment) Case-Control Study. Am. J. Clin. Nutr..

[203] Javed H., Azimullah S., Haque M.E., Ojha S.K. (2016). Cannabinoid Type 2 (CB2) Receptors Activation Protects against Oxidative Stress and Neuroinflammation Associated Dopaminergic Neurodegeneration in Rotenone Model of Parkinson’s Disease. Front. Neurosci..

[204] Zhao Y.-H., Fu H.-G., Cheng H., Zheng R.-J., Wang G., Li S., Li E.-Y., Li L.-G. (2022). Electroacupuncture at Zusanli Ameliorates the Autistic-like Behaviors of Rats through Activating the Nrf2-Mediated Antioxidant Responses. Gene.

[205] Morris G., Walder K., Berk M., Carvalho A.F., Marx W., Bortolasci C.C., Yung A.R., Puri B.K., Maes M. (2022). Intertwined Associations between Oxidative and Nitrosative Stress and Endocannabinoid System Pathways: Relevance for Neuropsychiatric Disorders. Prog. Neuro-Psychopharmacol. Biol. Psychiatry.

[206] Aran A., Eylon M., Harel M., Polianski L., Nemirovski A., Tepper S., Schnapp A., Cassuto H., Wattad N., Tam J. (2019). Lower Circulating Endocannabinoid Levels in Children with Autism Spectrum Disorder. Mol. Autism.

[207] Chakrabarti B., Persico A., Battista N., Maccarrone M. (2015). Endocannabinoid Signaling in Autism. Neurotherapeutics.

[208] Chełchowska M., Gajewska J., Szczepanik E., Mazur J., Cychol A., Kuźniar-Pałka A., Ambroszkiewicz J. (2025). Oxidative Stress Indicated by Nuclear Transcription Factor Nrf2 and Glutathione Status in the Blood of Young Children with Autism Spectrum Disorder: Pilot Study. Antioxidants.

[209] Ayaydin H., Akaltun İ., Koyuncu İ., Çelİk H., Kİrmİt A., Takatak H. (2020). High KEAP1, NRF2 and Low HO-1 Serum Levels in Children with Autism. Noro Psikiyatr. Ars..

[210] Subasi Turgut F., Karadag M., Taysi S., Hangül Z., Gokcen C. (2024). NRF2, KEAP1 and GSK-3 Levels in Autism Spectrum Disorder: A Case Control Study. Int. J. Dev. Disabil..

[211] Furnari M.A., Saw C.L.-L., Kong A.-N., Wagner G.C. (2014). Altered Behavioral Development in Nrf2 Knockout Mice Following Early Postnatal Exposure to Valproic Acid. Brain Res. Bull..

[212] Shah A., Varma M., Bhandari R. (2024). Exploring Sulforaphane as Neurotherapeutic: Targeting Nrf2-Keap & Nf-Kb Pathway Crosstalk in ASD. Metab. Brain Dis..

[213] Panes J.D., Wendt A., Ramirez-Molina O., Castro P.A., Fuentealba J. (2022). Deciphering the Role of PGC-1α in Neurological Disorders: From Mitochondrial Dysfunction to Synaptic Failure. Neural Regen. Res..

[214] Souder D.C., McGregor E.R., Rhoads T.W., Clark J.P., Porter T.J., Eliceiri K., Moore D.L., Puglielli L., Anderson R.M. (2023). Mitochondrial Regulator PGC-1a in Neuronal Metabolism and Brain Aging. bioRxiv.

[215] You W., Knoops K., Berendschot T.T.J.M., Benedikter B.J., Webers C.A.B., Reutelingsperger C.P.M., Gorgels T.G.M.F. (2024). PGC-1a Mediated Mitochondrial Biogenesis Promotes Recovery and Survival of Neuronal Cells from Cellular Degeneration. Cell Death Discov..

[216] Bam S., Buchanan E., Mahony C., O’Ryan C. (2021). DNA Methylation of PGC-1α Is Associated With Elevated MtDNA Copy Number and Altered Urinary Metabolites in Autism Spectrum Disorder. Front. Cell Dev. Biol..

[217] Ryter S.W., Otterbein L.E., Morse D., Choi A.M.K. (2002). Heme Oxygenase/Carbon Monoxide Signaling Pathways: Regulation and Functional Significance. Mol. Cell. Biochem..

[218] Augusto O., Truzzi D.R. (2021). Carbon Dioxide Redox Metabolites in Oxidative Eustress and Oxidative Distress. Biophys. Rev..

[219] Jo-Watanabe A., Inaba T., Osada T., Hashimoto R., Nishizawa T., Okuno T., Ihara S., Touhara K., Hattori N., Oh-Hora M. (2024). Bicarbonate Signalling via G Protein-Coupled Receptor Regulates Ischaemia-Reperfusion Injury. Nat. Commun..

[220] Whiteman M., Cheung N.S., Zhu Y.-Z., Chu S.H., Siau J.L., Wong B.S., Armstrong J.S., Moore P.K. (2005). Hydrogen Sulphide: A Novel Inhibitor of Hypochlorous Acid-Mediated Oxidative Damage in the Brain?. Biochem. Biophys. Res. Commun..

[221] Kimura H. (2002). Hydrogen Sulfide as a Neuromodulator. Mol. Neurobiol..

[222] Manivasagam T., Arunadevi S., Essa M.M., SaravanaBabu C., Borah A., Thenmozhi A.J., Qoronfleh M.W. (2020). Role of Oxidative Stress and Antioxidants in Autism. Adv. Neurobiol..

[223] Bonomini F., Siniscalco D., Schultz S., Carnovale C., Barthélémy C., Fazzi E.M. (2022). Editorial: Antioxidants in Autism Spectrum Disorders. Front. Psychiatry.

[224] Essa M.M., Braidy N., Waly M.I., Al-Farsi Y.M., Al-Sharbati M., Subash S., Amanat A., Al-Shaffaee M.A., Guillemin G.J. (2013). Impaired Antioxidant Status and Reduced Energy Metabolism in Autistic Children. Res. Autism Spectr. Disord..

[225] Mondal A., Mukherjee S., Dar W., Singh S., Pati S. (2021). Role of Glucose 6-Phosphate Dehydrogenase (G6PD) Deficiency and Its Association to Autism Spectrum Disorders. Biochimica et biophysica acta. Mol. Basis Dis..

[226] Nelson D.L., Cox M.M. (2013). Lehninger Principles of Biochemistry.

[227] Al-Salehi S.M., Al-Hifthy E.H., Ghaziuddin M. (2009). Autism in Saudi Arabia: Presentation, Clinical Correlates and Comorbidity. Transcult. Psychiatry.

[228] Jeng W., Loniewska M.M., Wells P.G. (2013). Brain Glucose-6-Phosphate Dehydrogenase Protects against Endogenous Oxidative DNA Damage and Neurodegeneration in Aged Mice. ACS Chem. Neurosci..

[229] Loniewska M.M., Gupta A., Bhatia S., MacKay-Clackett I., Jia Z., Wells P.G. (2020). DNA Damage and Synaptic and Behavioural Disorders in Glucose-6-Phosphate Dehydrogenase-Deficient Mice. Redox Biol..

[230] Al-Gadani Y., El-Ansary A., Attas O., Al-Ayadhi L. (2009). Metabolic Biomarkers Related to Oxidative Stress and Antioxidant Status in Saudi Autistic Children. Clin. Biochem..

[231] Chauhan A., Audhya T., Chauhan V. (2012). Brain Region-Specific Glutathione Redox Imbalance in Autism. Neurochem. Res..

[232] Gu F., Chauhan V., Chauhan A. (2015). Glutathione Redox Imbalance in Brain Disorders. Curr. Opin. Clin. Nutr. Metab. Care.

[233] Endres D., Tebartz van Elst L., Meyer S.A., Feige B., Nickel K., Bubl A., Riedel A., Ebert D., Lange T., Glauche V. (2017). Glutathione Metabolism in the Prefrontal Brain of Adults with High-Functioning Autism Spectrum Disorder: An MRS Study. Mol. Autism.

[234] Bowers K., Li Q., Bressler J., Avramopoulos D., Newschaffer C., Fallin M.D. (2011). Glutathione Pathway Gene Variation and Risk of Autism Spectrum Disorders. J. Neurodev. Disord..

[235] Hodgson N.W., Waly M.I., Al-Farsi Y.M., Al-Sharbati M.M., Al-Farsi O., Ali A., Ouhtit A., Zang T., Zhou Z.S., Deth R.C. (2014). Decreased Glutathione and Elevated Hair Mercury Levels Are Associated with Nutritional Deficiency-Based Autism in Oman. Exp. Biol. Med..

[236] Scapagnini G., Vasto S., Abraham N.G., Caruso C., Zella D., Fabio G. (2011). Modulation of Nrf2/ARE Pathway by Food Polyphenols: A Nutritional Neuroprotective Strategy for Cognitive and Neurodegenerative Disorders. Mol. Neurobiol..

[237] Porokhovnik L.N., Pisarev V.M., Chumachenko A.G., Chudakova J.M., Ershova E.S., Veiko N.N., Gorbachevskaya N.L., Mamokhina U.A., Sorokin A.B., Basova A.Y. (2023). Association of NEF2L2 Rs35652124 Polymorphism with Nrf2 Induction and Genotoxic Stress Biomarkers in Autism. Genes.

[238] Ming X., Johnson W.G., Stenroos E.S., Mars A., Lambert G.H., Buyske S. (2010). Genetic Variant of Glutathione Peroxidase 1 in Autism. Brain Dev..

[239] Chen L., Shi X.-J., Liu H., Mao X., Gui L.-N., Wang H., Cheng Y. (2021). Oxidative Stress Marker Aberrations in Children with Autism Spectrum Disorder: A Systematic Review and Meta-Analysis of 87 Studies (N = 9109). Transl. Psychiatry.

[240] Aoyama K. (2021). Glutathione in the Brain. Int. J. Mol. Sci..

[241] James S.J., Melnyk S., Jernigan S., Cleves M.A., Halsted C.H., Wong D.H., Cutler P., Bock K., Boris M., Bradstreet J.J. (2006). Metabolic Endophenotype and Related Genotypes Are Associated with Oxidative Stress in Children with Autism. Am. J. Med. Genet. Part B Neuropsychiatr. Genet..

[242] James S.J., Cutler P., Melnyk S., Jernigan S., Janak L., Gaylor D.W., Neubrander J.A. (2004). Metabolic Biomarkers of Increased Oxidative Stress and Impaired Methylation Capacity in Children with Autism. Am. J. Clin. Nutr..

[243] Lu J., Holmgren A. (2014). The Thioredoxin Antioxidant System. Free Radic. Biol. Med..

[244] Ren X., Zou L., Zhang X., Branco V., Wang J., Carvalho C., Holmgren A., Lu J. (2017). Redox Signaling Mediated by Thioredoxin and Glutathione Systems in the Central Nervous System. Antioxid. Redox Signal..

[245] Brigelius-Flohé R., Maiorino M. (2013). Glutathione Peroxidases. Biochim. Biophys. Acta.

[246] Savaskan N.E., Bräuer A.U., Kühbacher M., Eyüpoglu I.Y., Kyriakopoulos A., Ninnemann O., Behne D., Nitsch R. (2003). Selenium Deficiency Increases Susceptibility to Glutamate-Induced Excitotoxicity. FASEB J..

[247] Lee K.H., Cha M., Lee B.H. (2021). Crosstalk between Neuron and Glial Cells in Oxidative Injury and Neuroprotection. Int. J. Mol. Sci..

[248] Dawi J., Mohan A.S., Misakyan Y., Affa S., Gonzalez E., Hajjar K., Nikoghosyan D., Fardeheb S., Tuohino C., Venketaraman V. (2024). The Role of Oxidative Stress in TB Meningitis and Therapeutic Options. Diseases.

[249] Glorieux C., Calderon P.B. (2017). Catalase, a Remarkable Enzyme: Targeting the Oldest Antioxidant Enzyme to Find a New Cancer Treatment Approach. Biol. Chem..

[250] Moreno S., Mugnaini E., Cerù M.P. (1995). Immunocytochemical Localization of Catalase in the Central Nervous System of the Rat. J. Histochem. Cytochem..

[251] Schad A., Fahimi H.D., Völkl A., Baumgart E. (2003). Expression of Catalase MRNA and Protein in Adult Rat Brain: Detection by Nonradioactive in Situ Hybridization with Signal Amplification by Catalyzed Reporter Deposition (ISH-CARD) and Immunohistochemistry (IHC)/Immunofluorescence (IF). J. Histochem. Cytochem..

[252] Yenkoyan K., Harutyunyan H., Harutyunyan A. (2018). A Certain Role of SOD/CAT Imbalance in Pathogenesis of Autism Spectrum Disorders. Free Radic. Biol. Med..

[253] Wu Y.H., Hsieh H.L. (2022). Roles of Heme Oxygenase-1 in Neuroinflammation and Brain Disorders. Antioxidants.

[254] Ba X., Boldogh I. (2018). 8-Oxoguanine DNA Glycosylase 1: Beyond Repair of the Oxidatively Modified Base Lesions. Redox Biol..

[255] Shpyleva S., Ivanovsky S., de Conti A., Melnyk S., Tryndyak V., Beland F.A., James S.J., Pogribny I.P. (2014). Cerebellar Oxidative DNA Damage and Altered DNA Methylation in the BTBR T+tf/J Mouse Model of Autism and Similarities with Human Post Mortem Cerebellum. PLoS ONE.

[256] Markkanen E., Meyer U., Dianov G.L. (2016). DNA Damage and Repair in Schizophrenia and Autism: Implications for Cancer Comorbidity and Beyond. Int. J. Mol. Sci..

[257] Gaita L., Manzi B., Sacco R., Lintas C., Altieri L., Lombardi F., Pawlowski T.L., Redman M., Craig D.W., Huentelman M.J. (2010). Decreased Serum Arylesterase Activity in Autism Spectrum Disorders. Psychiatry Res..

[258] Paşca S.P., Nemeş B., Vlase L., Gagyi C.E., Dronca E., Miu A.C., Dronca M. (2006). High Levels of Homocysteine and Low Serum Paraoxonase 1 Arylesterase Activity in Children with Autism. Life Sci..

[259] D’Amelio M., Ricci I., Sacco R., Liu X., D’Agruma L., Muscarella L.A., Guarnieri V., Militerni R., Bravaccio C., Elia M. (2005). Paraoxonase Gene Variants Are Associated with Autism in North America, but Not in Italy: Possible Regional Specificity in Gene-Environment Interactions. Mol. Psychiatry.

[260] Dufault R., Lukiw W.J., Crider R., Schnoll R., Wallinga D., Deth R. (2012). A Macroepigenetic Approach to Identify Factors Responsible for the Autism Epidemic in the United States. Clin. Epigenet..

[261] Wang Y., Branicky R., Noë A., Hekimi S. (2018). Superoxide Dismutases: Dual Roles in Controlling ROS Damage and Regulating ROS Signaling. J. Cell Biol..

[262] Gulesserian T., Seidl R., Hardmeier R., Cairns N., Lubec G. (2001). Superoxide Dismutase SOD1, Encoded on Chromosome 21, but Not SOD2 Is Overexpressed in Brains of Patients with Down Syndrome. J. Investig. Med..

[263] Shareef A.A., Kheder A.H., Albarzinji N., Karim K.J., Smail S.W., Mahmood A.A., Amin K. (2025). Oxidative Markers and SOD Variant: Predictors of Autism Severity and Susceptibility. Future Sci. OA.

[264] Zhang T., Sun Y., Wei J., Zhao G., Hao W., Lv Z., Chen X., Liu Y., Wei F. (2023). Shorter Telomere Length in Children with Autism Spectrum Disorder Is Associated with Oxidative Stress. Front. Psychiatry.

[265] Vellingiri B., Venkatesan D., Iyer M., Mohan G., Krishnan P., Sai Krishna K., R S., Narayanasamy A., Gopalakrishnan A.V., Kumar N.S. (2023). Concurrent Assessment of Oxidative Stress and MT-ATP6 Gene Profiling to Facilitate Diagnosis of Autism Spectrum Disorder (ASD) in Tamil Nadu Population. J. Mol. Neurosci..

[266] Wang L., Jia J., Zhang J., Li K. (2016). Serum Levels of SOD and Risk of Autism Spectrum Disorder: A Case-Control Study. Int. J. Dev. Neurosci..

[267] Nadeem A., Ahmad S.F., Attia S.M., Al-Ayadhi L.Y., Al-Harbi N.O., Bakheet S.A. (2019). Dysregulated Enzymatic Antioxidant Network in Peripheral Neutrophils and Monocytes in Children with Autism. Prog. Neuro-Psychopharmacol. Biol. Psychiatry.

[268] Afrazeh M., Saedisar S., Khakzad M.R., Hojati M. (2015). Measurement of Serum Superoxide Dismutase and Its Relevance to Disease Intensity Autistic Children. Maedica.

[269] Yui K., Tanuma N., Yamada H., Kawasaki Y. (2017). Reduced Endogenous Urinary Total Antioxidant Power and Its Relation of Plasma Antioxidant Activity of Superoxide Dismutase in Individuals with Autism Spectrum Disorder. Int. J. Dev. Neurosci..

[270] Salari Z., Moslemizadeh A., Tezerji S.S., Sabet N., Parizi A.S., Khaksari M., Sheibani V., Jafari E., Shafieipour S., Bashiri H. (2024). Sex-Dependent Alterations of Inflammatory Factors, Oxidative Stress, and Histopathology of the Brain-Gut Axis in a VPA-Induced Autistic-like Model of Rats. Birth Defects Res..

[271] Yorbik O., Sayal A., Akay C., Akbiyik D.I., Sohmen T. (2002). Investigation of Antioxidant Enzymes in Children with Autistic Disorder. Prostaglandins Leukot. Essent. Fat. Acids.

[272] El-Ansary A., Bjørklund G., Chirumbolo S., Alnakhli O.M. (2017). Predictive Value of Selected Biomarkers Related to Metabolism and Oxidative Stress in Children with Autism Spectrum Disorder. Metab. Brain Dis..

[273] Bjørklund G., Zou L., Peana M., Chasapis C.T., Hangan T., Lu J., Maes M. (2022). The Role of the Thioredoxin System in Brain Diseases. Antioxidants.

[274] Rossignol D.A., Frye R.E. (2012). Mitochondrial Dysfunction in Autism Spectrum Disorders: A Systematic Review and Meta-Analysis. Mol. Psychiatry.

[275] Ornoy A., Reece E.A., Pavlinkova G., Kappen C., Miller R.K. (2015). Effect of Maternal Diabetes on the Embryo, Fetus, and Children: Congenital Anomalies, Genetic and Epigenetic Changes and Developmental Outcomes. Birth Defects Res. Part C Embryo Today Rev..

[276] Hollis F., Kanellopoulos A.K., Bagni C. (2017). Mitochondrial Dysfunction in Autism Spectrum Disorder: Clinical Features and Perspectives. Curr. Opin. Neurobiol..

[277] Al-Kafaji G., Jahrami H.A., Alwehaidah M.S., Alshammari Y., Husni M. (2023). Mitochondrial DNA Copy Number in Autism Spectrum Disorder and Attention Deficit Hyperactivity Disorder: A Systematic Review and Meta-Analysis. Front. Psychiatry.

[278] Kovacheva E., Gevezova M., Mehterov N., Kazakova M., Sarafian V. (2025). The Intersection of Mitophagy and Autism Spectrum Disorder: A Systematic Review. Int. J. Mol. Sci..

[279] Citrigno L., Muglia M., Qualtieri A., Spadafora P., Cavalcanti F., Pioggia G., Cerasa A. (2020). The Mitochondrial Dysfunction Hypothesis in Autism Spectrum Disorders: Current Status and Future Perspectives. Int. J. Mol. Sci..

[280] Frye R.E. (2020). Mitochondrial Dysfunction in Autism Spectrum Disorder: Unique Abnormalities and Targeted Treatments. Semin. Pediatr. Neurol..

[281] Sharon G., Sampson T.R., Geschwind D.H., Mazmanian S.K. (2016). The Central Nervous System and the Gut Microbiome. Cell.

[282] Frye R.E., Rossignol D.A., Scahill L., McDougle C.J., Huberman H., Quadros E.V. (2020). Treatment of Folate Metabolism Abnormalities in Autism Spectrum Disorder. Semin. Pediatr. Neurol..

[283] Berni Canani R., Di Costanzo M., Leone L. (2012). The Epigenetic Effects of Butyrate: Potential Therapeutic Implications for Clinical Practice. Clin. Epigenetics.

[284] Thomas R.H., Meeking M.M., Mepham J.R., Tichenoff L., Possmayer F., Liu S., MacFabe D.F. (2012). The Enteric Bacterial Metabolite Propionic Acid Alters Brain and Plasma Phospholipid Molecular Species: Further Development of a Rodent Model of Autism Spectrum Disorders. J. Neuroinflam..

[285] Fiorentino M., Sapone A., Senger S., Camhi S.S., Kadzielski S.M., Buie T.M., Kelly D.L., Cascella N., Fasano A. (2016). Blood-Brain Barrier and Intestinal Epithelial Barrier Alterations in Autism Spectrum Disorders. Mol. Autism.

[286] Richardson L.A. (2017). Evolving as a Holobiont. PLoS Biol..

[287] Modafferi S., Lupo G., Tomasello M., Rampulla F., Ontario M., Scuto M., Salinaro A.T., Arcidiacono A., Anfuso C.D., Legmouz M. (2024). Antioxidants, Hormetic Nutrition, and Autism. Curr. Neuropharmacol..

[288] Sterling K.G., Dodd G.K., Alhamdi S., Asimenios P.G., Dagda R.K., De Meirleir K.L., Hudig D., Lombardi V.C. (2022). Mucosal Immunity and the Gut-Microbiota-Brain-Axis in Neuroimmune Disease. Int. J. Mol. Sci..

[289] Pridmore R.D., Pittet A.-C., Praplan F., Cavadini C. (2008). Hydrogen Peroxide Production by Lactobacillus Johnsonii NCC 533 and Its Role in Anti-Salmonella Activity. FEMS Microbiol. Lett..

[290] Johnson S.L., Kirk R.D., DaSilva N.A., Ma H., Seeram N.P., Bertin M.J. (2019). Polyphenol Microbial Metabolites Exhibit Gut and Blood−Brain Barrier Permeability and Protect Murine Microglia against LPS-Induced Inflammation. Metabolites.

[291] Vauzour D. (2012). Dietary Polyphenols as Modulators of Brain Functions: Biological Actions and Molecular Mechanisms Underpinning Their Beneficial Effects. Oxidative Med. Cell. Longev..

[292] Stilling R.M., van de Wouw M., Clarke G., Stanton C., Dinan T.G., Cryan J.F. (2016). The Neuropharmacology of Butyrate: The Bread and Butter of the Microbiota-Gut-Brain Axis?. Neurochem. Int..

[293] Fatemi S.H., Halt A.R., Stary J.M., Kanodia R., Schulz S.C., Realmuto G.R. (2002). Glutamic Acid Decarboxylase 65 and 67 KDa Proteins Are Reduced in Autistic Parietal and Cerebellar Cortices. Biol. Psychiatry.

[294] Horder J., Petrinovic M.M., Mendez M.A., Bruns A., Takumi T., Spooren W., Barker G.J., Künnecke B., Murphy D.G. (2018). Glutamate and GABA in Autism Spectrum Disorder—A Translational Magnetic Resonance Spectroscopy Study in Man and Rodent Models. Transl. Psychiatry.

[295] Carpita B., Nardi B., Palego L., Cremone I.M., Massimetti G., Carmassi C., Betti L., Giannaccini G., Dell’Osso L. (2023). Kynurenine Pathway and Autism Spectrum Phenotypes: An Investigation among Adults with Autism Spectrum Disorder and Their First-Degree Relatives. CNS Spectr..

[296] Almulla A.F., Thipakorn Y., Zhou B., Vojdani A., Paunova R., Maes M. (2024). The Tryptophan Catabolite or Kynurenine Pathway in Long COVID Disease: A Systematic Review and *meta*-Analysis. Neuroscience.

[297] Yip J., Soghomonian J.-J., Blatt G.J. (2007). Decreased GAD67 MRNA Levels in Cerebellar Purkinje Cells in Autism: Pathophysiological Implications. Acta Neuropathol..

[298] Jin F., Wang Z. (2024). Mapping the Structure of Biomarkers in Autism Spectrum Disorder: A Review of the Most Influential Studies. Front. Neurosci..

[299] Anashkina A.A., Erlykina E.I. (2021). Molecular Mechanisms of Aberrant Neuroplasticity in Autism Spectrum Disorders (Review). Sovrem. Tekhnologii Meditsine.

[300] Estes M.L., McAllister A.K. (2016). Maternal Immune Activation: Implications for Neuropsychiatric Disorders. Science.

[301] Al-Harbi N.O., Nadeem A., Ahmad S.F., Al-Ayadhi L.Y., Al-Harbi M.M., As Sobeai H.M., Ibrahim K.E., Bakheet S.A. (2020). Elevated Expression of Toll-like Receptor 4 Is Associated with NADPH Oxidase-Induced Oxidative Stress in B Cells of Children with Autism. Int. Immunopharmacol..

[302] Abdel-Haq M., Ojha S.K., Hamoudi W., Kumar A., Tripathi M.K., Khaliulin I., Domb A.J., Amal H. (2023). Effects of Extended-Release 7-Nitroindazole Gel Formulation Treatment on the Behavior of Shank3 Mouse Model of Autism. Nitric Oxide.

[303] Hughes H.K., Moreno R.J., Ashwood P. (2023). Innate Immune Dysfunction and Neuroinflammation in Autism Spectrum Disorder (ASD). Brain Behav. Immun..

[304] Xu H., Jiang J., Chen W., Li W., Chen Z. (2019). Vascular Macrophages in Atherosclerosis. J. Immunol. Res..

[305] Martínez de Toda I., Ceprián N., Díaz-Del Cerro E., De la Fuente M. (2021). The Role of Immune Cells in Oxi-Inflamm-Aging. Cells.

[306] Meyer U. (2014). Prenatal Poly(I:C) Exposure and Other Developmental Immune Activation Models in Rodent Systems. Biol. Psychiatry.

[307] Choi G.B., Yim Y.S., Wong H., Kim S., Kim H., Kim S.V., Hoeffer C.A., Littman D.R., Huh J.R. (2016). The Maternal Interleukin-17a Pathway in Mice Promotes Autism-like Phenotypes in Offspring. Science.

[308] Xu X., Tan L., Zhang X. (2025). Prenatal Exposure to Valproic Acid may Alter CD200/CD200R Signaling Pathways in a Rat Model of Autism Spectrum Disorder. Alpha Psychiatry.

[309] Li Q., Barres B.A. (2018). Microglia and Macrophages in Brain Homeostasis and Disease. Nat. Rev. Immunol..

[310] Ndrepepa G. (2019). Myeloperoxidase—A Bridge Linking Inflammation and Oxidative Stress with Cardiovascular Disease. Clin. Chim. Acta.

[311] Kalliolias G.D., Ivashkiv L.B. (2016). TNF Biology, Pathogenic Mechanisms and Emerging Therapeutic Strategies. Nat. Rev. Rheumatol..

[312] Mittal M., Siddiqui M.R., Tran K., Reddy S.P., Malik A.B. (2014). Reactive Oxygen Species in Inflammation and Tissue Injury. Antioxid. Redox Signal..

[313] Biswas S.K. (2016). Does the Interdependence between Oxidative Stress and Inflammation Explain the Antioxidant Paradox?. Oxidative Med. Cell. Longev..

[314] Pivtoraiko V.N., Stone S.L., Roth K.A., Shacka J.J. (2009). Oxidative stress and autophagy in the regulation of lysosome-dependent neuron death. Antioxid. Redox Signal..

[315] Finkbeiner S. (2020). The Autophagy Lysosomal Pathway and Neurodegeneration. Cold Spring Harb. Perspect. Biol..

[316] Zhang J., Zhang J.-X., Zhang Q.-L. (2016). PI3K/AKT/MTOR-Mediated Autophagy in the Development of Autism Spectrum Disorder. Brain Res. Bull..

[317] Chaudry S., Vasudevan N. (2022). mTOR-Dependent Spine Dynamics in Autism. Front. Mol. Neurosci..

[318] Switon K., Kotulska K., Janusz-Kaminska A., Zmorzynska J., Jaworski J. (2017). Molecular neurobiology of mTOR. Neuroscience.

[319] Tang G., Gudsnuk K., Kuo S.H., Cotrina M.L., Rosoklija G., Sosunov A., Sonders M.S., Kanter E., Castagna C., Yamamoto A. (2014). Loss of mTOR-dependent macroautophagy causes autistic-like synaptic pruning deficits. Neuron.

[320] Zapata-Muñoz J., Villarejo-Zori B., Largo-Barrientos P., Boya P. (2021). Towards a better understanding of the neuro-developmental role of autophagy in sickness and in health. Cell Stress.

[321] Ganesan H., Balasubramanian V., Iyer M., Venugopal A., Subramaniam M.D., Cho S.G., Vellingiri B. (2019). mTOR signalling pathway—A root cause for idiopathic autism?. BMB Rep..

[322] Berger J., Dorninger F., Forss-Petter S., Kunze M. (2016). Peroxisomes in brain development and function. Biochim. Biophys. Acta.

[323] Altun H., Şahin N., Kurutaş E.B., Karaaslan U., Sevgen F.H., Fındıklı E. (2018). Assessment of malondialdehyde levels, superoxide dismutase, and catalase activity in children with autism spectrum disorders. Psychiatry Clin. Psychopharmacol..

[324] Cao S.S., Kaufman R.J. (2014). Endoplasmic Reticulum Stress and Oxidative Stress in Cell Fate Decision and Human Disease. Antioxid. Redox Signal..

[325] Singh R., Kaur N., Choubey V., Dhingra N., Kaur T. (2024). Endoplasmic Reticulum Stress and Its Role in Various Neurodegenerative Diseases. Brain Res..

[326] Wilson C., González-Billault C. (2015). Regulation of cytoskeletal dynamics by redox signaling and oxidative stress: Implications for neuronal development and trafficking. Front. Cell. Neurosci..

[327] Livanos P., Galatis B., Apostolakos P. (2014). The interplay between ROS and tubulin cytoskeleton in plants. Plant Signal. Behav..

[328] Oh C.K., Nakamura T., Zhang X., Lipton S.A. (2024). Redox regulation, protein S-nitrosylation, and synapse loss in Alzheimer’s and related dementias. Neuron.

[329] Landino L.M., Hasan R., McGaw A., Cooley S., Smith A.W., Masselam K., Kim G. (2002). Peroxynitrite oxidation of tubulin sulfhydryls inhibits microtubule polymerization. Arch. Biochem. Biophys..

[330] Gilbert J., Man H.Y. (2017). Fundamental Elements in Autism: From Neurogenesis and Neurite Growth to Synaptic Plasticity. Front. Cell. Neurosci..

[331] Oswald M.C., Brooks P.S., Zwart M.F., Mukherjee A., West R.J., Giachello C.N., Morarach K., Baines R.A., Sweeney S.T., Landgraf M. (2018). Reactive oxygen species regulate activity-dependent neuronal plasticity in Drosophila. eLife.

[332] Sanders S.J., Ercan-Sencicek A.G., Hus V., Luo R., Murtha M.T., Moreno-De-Luca D., Chu S.H., Moreau M.P., Gupta A.R., Thomson S.A. (2011). Multiple Recurrent de Novo CNVs, Including Duplications of the 7q11.23 Williams Syndrome Region, Are Strongly Associated with Autism. Neuron.

[333] Tang G., Gutierrez Rios P., Kuo S.-H., Akman H.O., Rosoklija G., Tanji K., Dwork A., Schon E.A., Dimauro S., Goldman J. (2013). Mitochondrial Abnormalities in Temporal Lobe of Autistic Brain. Neurobiol. Dis..

[334] Napoli E., Wong S., Giulivi C. (2013). Evidence of Reactive Oxygen Species-Mediated Damage to Mitochondrial DNA in Children with Typical Autism. Mol. Autism.

[335] Landrigan P.J. (2010). What Causes Autism? Exploring the Environmental Contribution. Curr. Opin. Pediatr..

[336] Oudin A., Frondelius K., Haglund N., Källén K., Forsberg B., Gustafsson P., Malmqvist E. (2019). Prenatal Exposure to Air Pollution as a Potential Risk Factor for Autism and ADHD. Environ. Int..

[337] Lyall K., Schmidt R.J., Hertz-Picciotto I. (2014). Maternal Lifestyle and Environmental Risk Factors for Autism Spectrum Disorders. Int. J. Epidemiol..

[338] Cocchia N., Ciani F., Tafuri S., Iorio E.L., Landolfi F., Ahmad R. (2016). Redoxomics and Oxidative Stress: From the Basic Research to the Clinical Practice. Free Radicals and Diseases.

[339] Lei X., Xie X.N., Yang J.X., Li Y.M. (2024). The emerging role of extracellular vesicles in the diagnosis and treatment of autism spectrum disorders. Psychiatry Res..

[340] Duarte A.C., Costa A.R., Gonçalves I., Quintela T., Preissner R., Santos C.R.A. (2022). The druggability of bitter taste receptors for the treatment of neurodegenerative disorders. Biochem. Pharmacol..

[341] Dobrachinski F., Ribeiro K.A., Bezerra I.C., da Silva A.J., Pereira C.M.M., Vellasques K., Padilha H.A., Haas S.E., Ávila D.S., Gubert P. (2025). Nutraceutical approaches for Autism Spectrum Disorder treatment. Behav. Brain Res..

[342] Hamilton C., Liebert A., Pang V., Magistretti P., Mitrofanis J. (2022). Lights on for Autism: Exploring Photobiomodulation as an Effective Therapeutic Option. Neurol. Int..

